# Tescalcin is a phagocytic checkpoint driving immune escape and limiting immunotherapeutic efficacy in hepatocellular carcinoma

**DOI:** 10.1172/JCI200415

**Published:** 2026-04-02

**Authors:** Jiong-Liang Wang, Jun-Cheng Wang, Yangxun Pan, Minrui He, Zhikai Zheng, Hao Zou, Tianqing Wu, Yuhan Zhang, Zili Hu, Yizhen Fu, Wei Peng, Zhenyun Yang, Li Xu, Yao-Jun Zhang, Min-Shan Chen, Dandan Hu, Jinbin Chen, Ming Zhao, Dong-Ping Chen, Zhong-Guo Zhou

**Affiliations:** 1 State Key Laboratory of Oncology in Southern China, Collaborative Innovation Center for Cancer Medicine, Department of Liver Surgery, Sun Yat-sen University Cancer Center, Guangzhou, China.; 2 Guangdong Province Key Laboratory of Pharmaceutical Functional Genes, MOE Key Laboratory of Gene Function and Regulation, School of Life Sciences, Sun Yat-sen University, Guangzhou, China.; 3 State Key Laboratory of Oncology in Southern China, Collaborative Innovation Center for Cancer Medicine, Department Minimally Invasive Interventional Therapy, Sun Yat-sen University Cancer Center, Guangzhou, China.; 4 Department of Radiation Oncology, The First Affiliated Hospital of Sun Yat-sen University, Guangzhou, China.

**Keywords:** Hepatology, Immunology, Oncology, Cancer immunotherapy, Liver cancer, Macrophages

## Abstract

Immunotherapies achieve durable responses in several cancers but show limited efficacy in refractory hepatocellular carcinoma (HCC). The mechanisms by which hepatoma cells evade immune recognition and limit immune checkpoint blockade (ICB) efficacy are incompletely defined. Here, we identified tumor-intrinsic tescalcin (TESC) as a previously unrecognized phagocytic checkpoint that contributes to immune evasion and ICB resistance in HCC. Mechanistically, H3K4 methylation drove TESC expression in hepatoma cells, facilitating cytosolic Ca^2+^ buffering and attenuating endoplasmic reticulum (ER) stress–induced calreticulin (CALR) plasma membrane exposure, an essential “eat-me” signal. Consequently, this process abrogated membrane CALR-directed phagocytosis by antigen-presenting cells (APCs), including macrophages and DCs, thereby impairing antigen presentation and subsequent T cell activation. Clinically, we found that elevated H3K4me3-TESC signaling was a promising prognostic biomarker for a poor ICB response in HCC. Importantly, in vivo disruption of this axis restored APC phagocytic function and enhanced the antitumor effects of ICB therapy. Therefore, targeting TESC-driven immune escape and its underlying epigenetic regulation may restore APC function and offer a precise therapeutic strategy to enhance immunotherapeutic efficacy in HCC.

## Abstract 2

Tumor-intrinsic tescalcin (TESC) is a previously unrecognized phagocytic checkpoint that contributes to immune evasion and ICB resistance in HCC.

## Graphical abstract

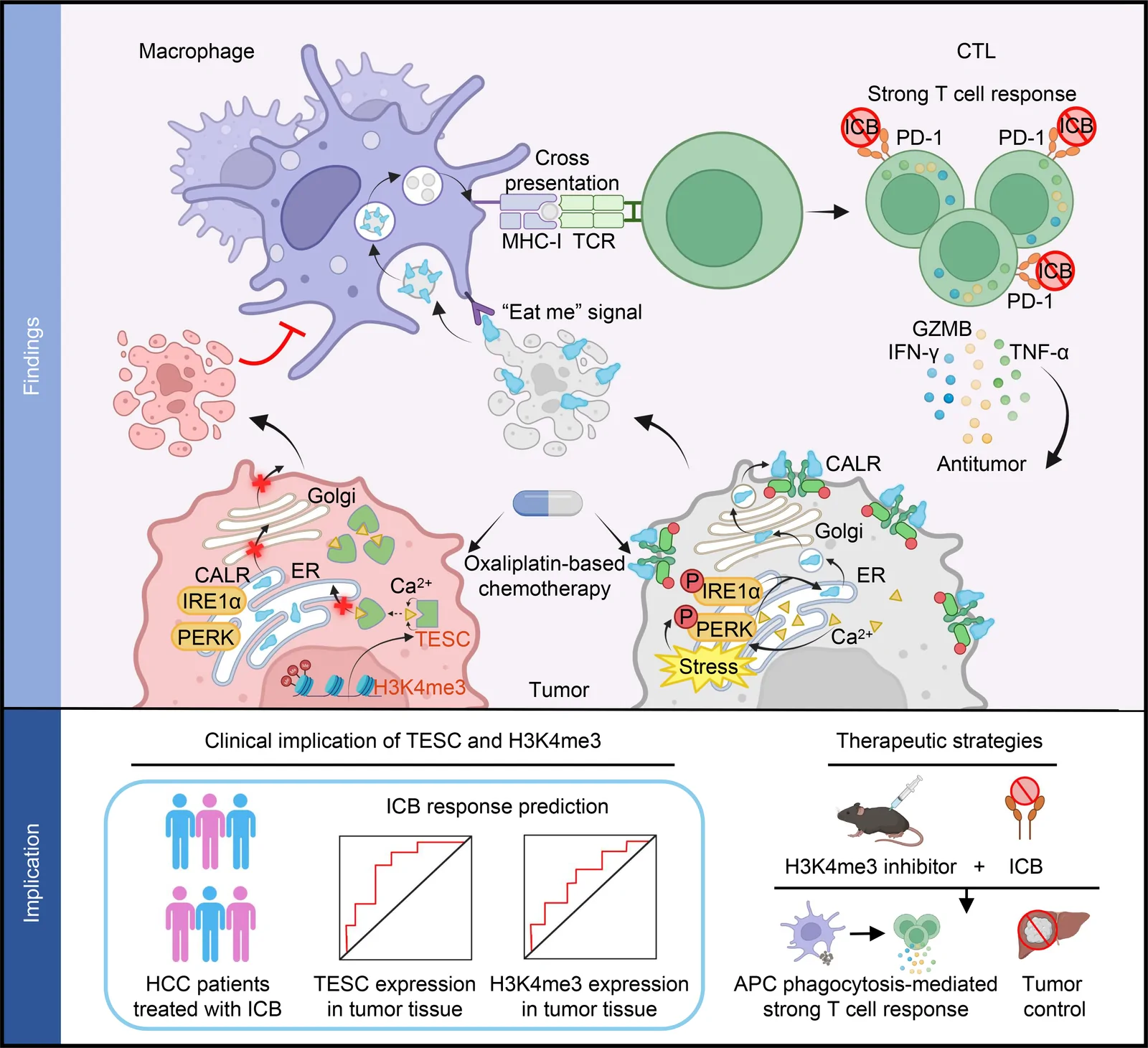

## Introduction

Immune checkpoint blockade (ICB) therapy has revolutionized cancer treatment by restoring and amplifying T cell–mediated antitumor responses (1–4). However, the majority of patients fail to benefit from ICB, owing to the complex interactions between tumor cells and the surrounding immune microenvironment (5–7). Elucidating these interactions is critical for identifying the cellular and molecular determinants of immune evasion, revealing potential therapeutic targets, and guiding the development of rational combination strategies. While tumor-intrinsic genetic and epigenetic alterations, such as DNA methylation, histone modifications, and chromatin remodeling, impair IFN-γ signaling, antigen presentation, and T cell effector function (8–11), how these changes affect upstream processes such as phagocytosis by antigen-presenting cells (APCs) remains poorly defined.

APCs prime and activate tumor-associated antigen–specific (TAA-specific) T cells and are critical for defining the success of checkpoint therapy (12). This process depends on whether APCs, including macrophages and DCs, can efficiently capture antigens from dead tumor cells via phagocytosis, present sufficient antigens to T cells, and activate T cells (13, 14). However, cancer cells exploit a suite of immune-evasive strategies by upregulating “don’t eat me” signals, including CD47, CD24, and MHC-I, to subvert phagocytic clearance (15). In parallel, tumor-associated macrophages (TAMs) often adopt immunosuppressive phenotypes that blunt antigen uptake and presentation. Clinically, the blockade of phagocytic checkpoints, including the CD47/SIRPα and CD24/Siglec-10 axes, has shown promise in restoring APC-mediated phagocytosis and enhancing the ICB response (16–18). In addition to suppressing inhibitory signals, tumors may also regulate prophagocytic “eat-me” cues, such as calreticulin (CALR), a key signal required for efficient APC-mediated uptake by dying tumor cells (19). Despite increasing recognition of the role of CALR in immunogenic clearance, the upstream regulatory mechanisms governing its surface expression in human cancer cells remain elusive. Elucidating these regulatory networks may reveal previously unrecognized axes of immune evasion and inform therapeutic approaches aimed at increasing sensitivity to ICB.

Hepatocellular carcinoma (HCC) usually occurs in inflamed fibrotic and/or cirrhotic livers with extensive leukocyte infiltration, where the inflammatory status in the tumor milieu profoundly shapes tumor cell behavior and modulates therapeutic responses (20–24). In this study, we identified tescalcin (TESC) as a previously unrecognized phagocytic checkpoint that contributes to immune evasion and ICB resistance. Mechanistically, the EF-hand calcium-binding protein TESC, which was intrinsically expressed by hepatoma cells driven by H3K4 methylation, buffered cytosolic Ca^2+^ and suppressed endoplasmic reticulum (ER) stress, thereby inhibiting CALR translocation to the plasma membrane. The resulting reduction in surface CALR expression impaired APC-mediated phagocytosis, suppressed antigen presentation, and attenuated T cell activation, ultimately resulting in immune evasion and ICB resistance. In addition, impeding H3K4 methylation-driven TESC expression in vivo restored phagocytic function and enhanced the therapeutic efficacy of ICB, revealing a therapeutically actionable mechanism of immune resistance in HCC.

## Results

### Tumor-intrinsic TESC contributes to resistance to antitumor immunity and immunotherapy.

**Figure 1:**
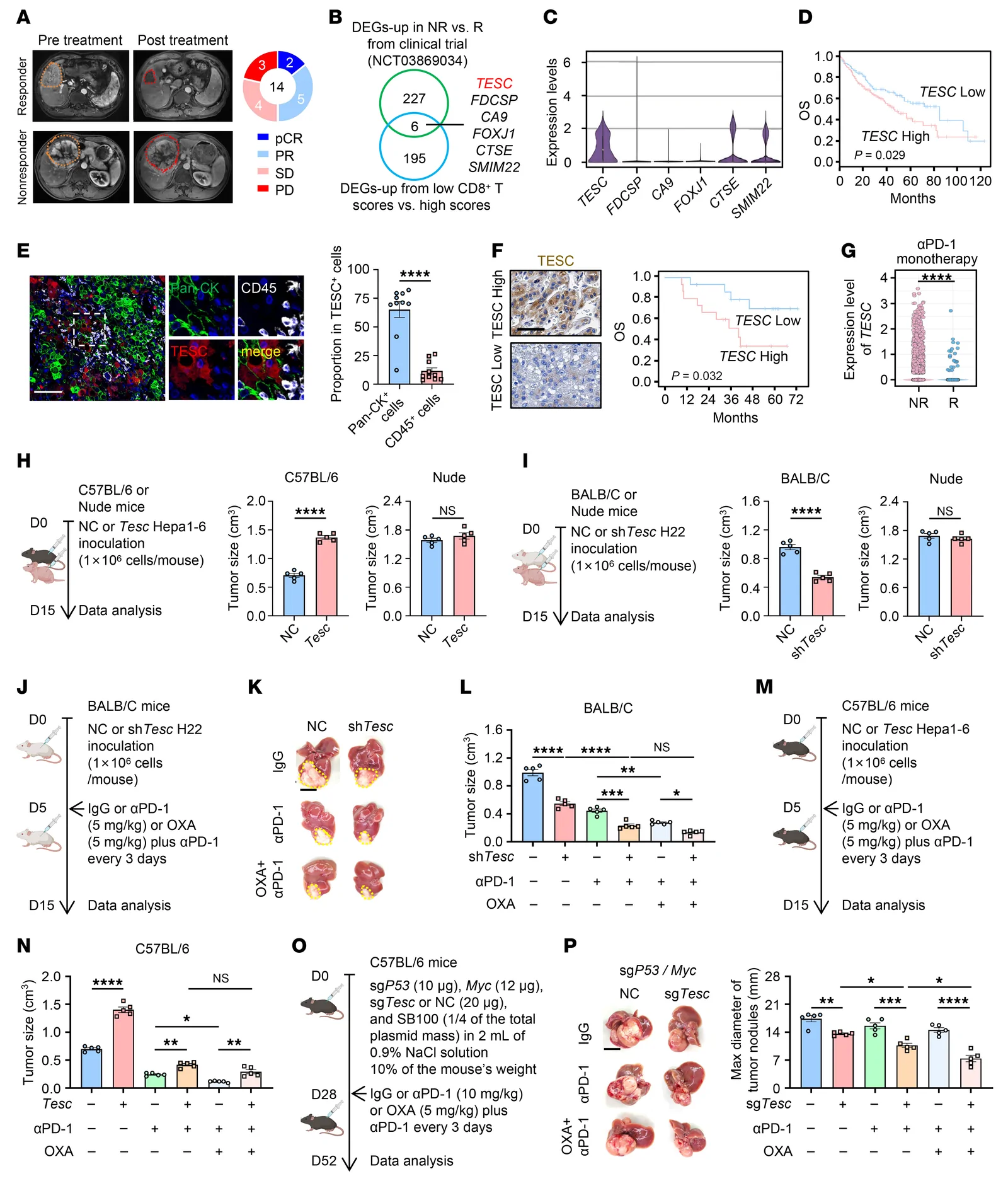
Tumor-intrinsic TESC contributes to resistance to antitumor immunity and immunotherapy. (**A**) Representative MRI enhancement scans of patients with HCC before and after OXA-based chemotherapy plus ICB (cohort 1, NCT03869034). (**B**) Upregulated genes in nonresponders from cohort 1 and both low CD8^+^ T cell infiltration scores and upregulated genes in HCC tissue from cohort 2 (TCGA) were determined. (**C**) Candidate gene expression in tumor cells from patients with HCC (GEO: GSE166635). (**D**) Correlations between TESC expression and OS in TCGA HCC patients grouped by median TESC expression. *P* = 0.029, by log-rank test. (**E**) Representative images of immunofluorescence for pan-CK, CD45, and TESC in HCC tissues (*n* = 20). Scale bar: 100 μm. Original magnification,×4. ****P < 0.0001, by 2-tailed Student’s *t* test. (**F**) Correlation of TESC level with OS of 30 patients with HCC treated with OXA-based chemotherapy plus ICB (cohort 3). Patients were grouped according to median TESC expression levels. Scale bar: 50 μm. *P* = 0.032, by log-rank test. (**G**) Transcript levels of TESC in HCC tumor cells from ICB responders and nonresponders (Mendeley Data, skrx2fz79n). ****P < 0.0001, by 2-tailed Student’s *t* test. (**H** and **I**) TESC-overexpressing (*Tesc*) Hepa1-6 and TESC-silenced (sh*Tesc*) H22 cells were inoculated into the livers of C57BL/6 or BALB/C and nude mice, respectively (*n* = 5). ****P < 0.0001, by 2-tailed Student’s *t* test. (**J**–**N**) Sh*Tesc* H22 (**J**–**L**) or *Tesc* Hepa1-6 hepatoma–bearing mice (**M** and **N**) were treated as described above (*n* = 5). Scale bar: 1 cm. *P < 0.05, **P < 0.01, ***P < 0.001, and ****P < 0.0001, by 1-way ANOVA. (**O** and **P**) C57BL/6 mice with spontaneous hepatomas were treated as described above (*n* = 5). Scale bar: 1 cm. *P < 0.05, **P < 0.01, ***P < 0.001, and ****P < 0.0001, by 1way ANOVA. All data are presented as the mean ± SEM. NR, nonresponder; pCR, pathological complete response; PD, progressive disease; PR, partial response; R, responder; SD, stable disease; αPD-1, anti–PD-1 antibody.

In our prospective clinical trial (NCT03869034) for locally advanced HCC, oxaliplatin-based (OXA-based) chemotherapy combined with anti–programmed cell death 1 (anti–PD-1) therapy elicited heterogeneous responses: 48.3% of patients achieved substantial tumor regression according to Response Evaluation Criteria in Solid Tumors (RECIST) 1.1 criteria, whereas others derived limited benefit or exhibited disease progression (Figure 1A). To investigate the mechanisms of resistance and uncover potential therapeutic targets, we performed transcriptomics profiling of tumors from 7 responders and 7 nonresponders. Integrated with The Cancer Genome Atlas (TCGA) data on CD8^+^ T cell infiltration identified 6 genes consistently upregulated in nonresponding tumors (Figure 1B). Among these, TESC emerged as the top candidate, given its high expression in malignant cells and strong inverse correlation with patient survival (Figure 1, C and D). The remaining candidates were either expressed at low levels or lacked association with clinical outcomes (Figure 1C and Supplemental Figure 1A; supplemental material available online with this article; https://doi.org/10.1172/JCI200415DS1). Multiplex immunofluorescence and single-cell RNA-seq (scRNA-seq) (GSE166635) demonstrated that TESC was predominantly expressed in tumor cells rather than immune cells (Figure 1E and Supplemental Figure 1B). Importantly, ROC analysis revealed that TESC expression exhibited robust predictive performance for response to OXA-based chemotherapy plus sintilimab (AUC = 0.88, *P* < 0.05) (Supplemental Figure 1C). Consistent with this observation, elevated TESC expression in this same cohort was significantly associated with shorter overall survival (OS) by Kaplan-Meier and Cox regression analyses (Figure 1F) and with reduced response rates to ICB therapy (Supplemental Figure 1D). These associations were independently validated using scRNA-seq data (Mendeley Data: skrx2fz79n), which confirmed a correlation between high TESC expression and diminished efficacy of anti–PD-1 therapy (Figure 1G). Notably, in contrast to its consistent association with immunotherapeutic outcomes, TESC expression showed no significant relationship with treatment response in an independent cohort of patients with HCC receiving OXA-based chemotherapy alone, despite a nonsignificant trend toward higher TESC levels in nonresponders (Supplemental Figure 1E). Collectively, these findings suggest that TESC may play a deleterious role in antitumor immunity and immunotherapeutic response rather than chemotherapy sensitivity.

We engineered Hepa1-6 cells to overexpress TESC and used an shRNA to silence TESC in H22 cells to investigate the function of TESC (Supplemental Figure 1, F–H). These cells were orthotopically implanted into immunocompetent C57BL/6, BALB/C or immunodeficient nude mice (Figure 1, H and I). In immunocompetent mice, TESC overexpression significantly accelerated Hepa1-6 hepatoma growth and shortened OS, whereas TESC silencing markedly inhibited H22 hepatoma progression and prolonged survival (Figure 1, H and I, and Supplemental Figure 1, I and J). In contrast, manipulation of TESC, either by silencing or overexpression, did not affect hepatoma growth in nude mice (Figure 1, H and I), indicating that TESC exerted its protumorigenic effects through a T cell–dependent mechanism. Consistent with this finding, anti–PD-1 therapy suppressed tumor growth in mice bearing either Hepa1-6 or H22 hepatoma, and this effect was enhanced by TESC silencing but attenuated by TESC overexpression (Figure 1, J–N). Notably, although anti–PD-1 therapy showed no therapeutic efficacy in the sg*P53*/*Myc*-driven spontaneous hepatoma model, TESC KO sensitized tumors to anti–PD-1 therapy, thereby substantially suppressing tumor development (Figure 1, O and P). Additionally, silencing or overexpressing TESC had little effect on the growth of H22 or Hepa1-6 cells in vitro (Supplemental Figure 1, K and L), and the addition of OXA did not confer further synergistic benefit when combined with TESC silencing and anti–PD-1 treatment (Figure 1, J–P). Thus, tumor-derived TESC may function as an intrinsic resistance mechanism of spontaneous and immunotherapy-induced tumor immunity.

### Tumor TESC impairs CD8^+^ T cell–mediated antitumor immunity.

**Figure 2:**
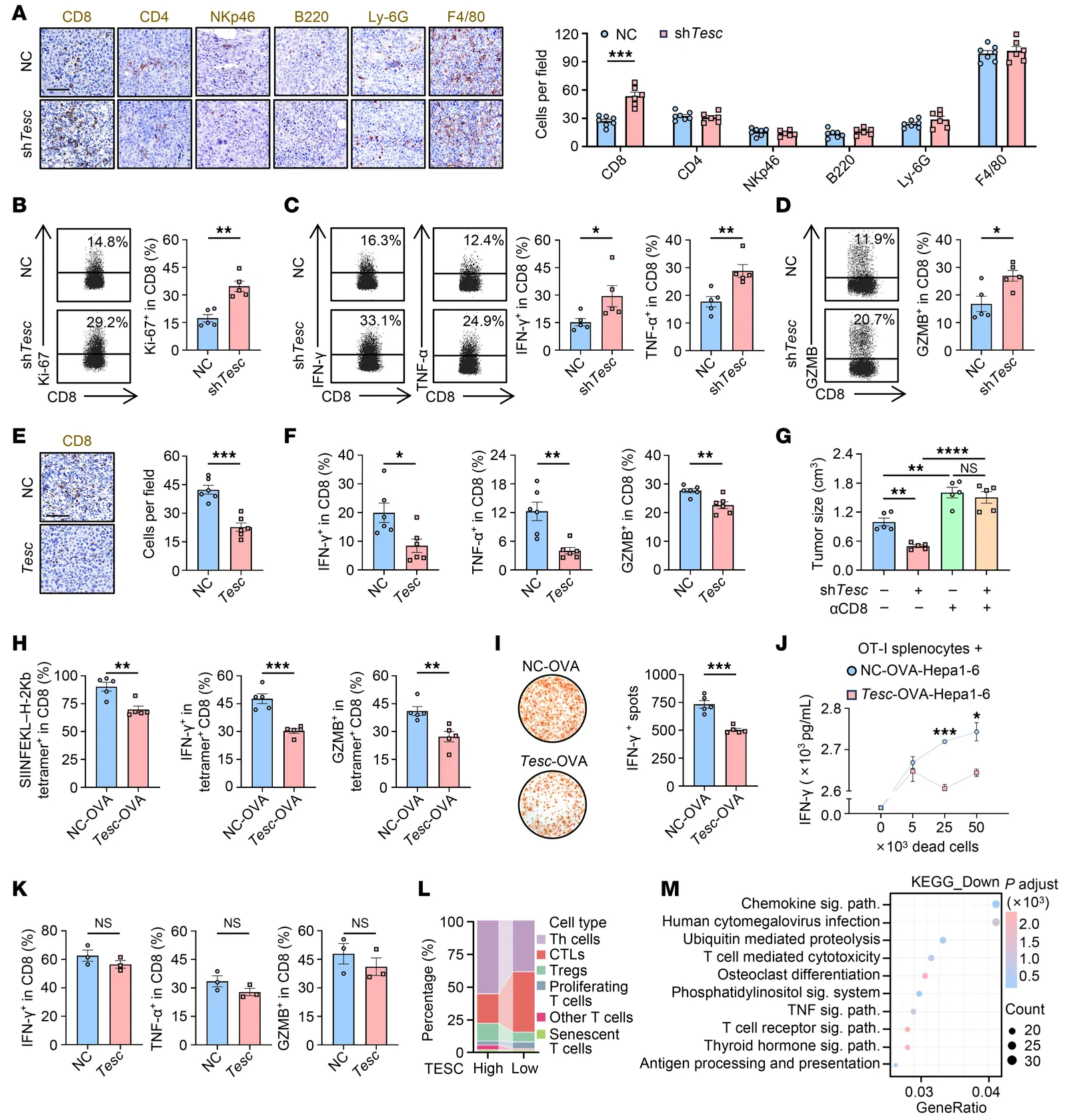
Tumor TESC impairs CD8^+^ T cell–mediated antitumor immunity. (**A**–**D**) sh*Tesc* H22 cells were inoculated into mouse livers (*n* = 5–7). The infiltration of CTLs (CD8^+^), Th cells (CD4^+^), NK cells (NKp46^+^), B cells (B220^+^), neutrophils (Ly-6G^+^), and macrophages (F4/80^+^) (**A**) and CTL functions (**B**–**D**) were analyzed. Scale bar: 50 μm. *P < 0.05, **P < 0.01, and ***P < 0.001, by 2-tailed Student’s *t* test. (**E** and **F**) *Tesc* Hepa1-6 cells were inoculated into mouse livers (*n* = 6). The infiltration of CTLs (CD8^+^) (**E**) and CTL functions (**F**) were analyzed. Scale bar: 50 μm. *P < 0.05, **P < 0.01, and ***P < 0.001, by 2-tailed Student’s *t* test. (**G**) Effects of CD8^+^ T cell depletion on sh*Tesc* in mouse hepatoma tissues (*n* = 5). **P < 0.01 and ****P < 0.0001, by 1-way ANOVA. (**H**) OVA-loaded *Tesc* Hepa1-6 cells were inoculated into the livers of C57BL/6 mice (*n* = 5). Surface expression of the SIINFEKL–H-2Kb tetramer in CTLs and the functions of tetramer^+^ CTLs were analyzed. **P < 0.01 and ***P < 0.001, by 2-tailed Student’s *t* test. (**I**) IFN-γ ELISPOT assay of tumor-infiltrating lymphocytes from mice bearing an OVA-loaded NC or *Tesc* Hepa1-6 hepatoma (*n* = 5). ***P < 0.001, by 2-tailed Student’s *t* test. (**J**) OT-I splenocytes were cultured with dead OVA-loaded *Tesc* Hepa1-6 cells for 3 days. IFN-γ production in the supernatant was measured by ELISA (*n* = 3). *P < 0.05 and ***P < 0.001, by 2-tailed Student’s *t* test. (**K**) *Tesc* Hepa1-6 cells were cultured with T cells in the presence of anti-CD3 and anti-CD28 antibodies. CTL function was analyzed (*n* = 3). Significance was determined by 2-tailed Student’s *t* test. (**L**) Comparison of T cell constitution between 2 groups (GSE151530). Patients were grouped by median tumor TESC expression levels in tumor cells. (**M**) The top 10 downregulated KEGG pathways enriched in T cells from tumor samples with high TESC expression. *P* adjust, adjusted *P* value; Down, downregulated. The Benjamini-Hochberg correction was applied. All data are presented as the mean ± SEM.

To elucidate the immunological basis for TESC-mediated immune evasion, we assessed immune cell infiltration and function in TESC-manipulated tumors. While the modulation of TESC expression did not alter tumor growth in nude mice, it profoundly influenced tumor progression in immunocompetent hosts (Figure 1, H and I), suggesting a T cell–dependent mechanism. In support of these findings, TESC silencing in hepatoma led to increased infiltration of CD8^+^ T cells, whereas other immune cell populations remained largely unchanged (Figure 2A). The flow cytometric analysis further revealed that CD8^+^ T cells in TESC-deficient tumors had an increased proliferative capacity and higher expression of effector molecules, including IFN-γ, TNF-α, and granzyme B (GZMB) (Figure 2, B–D). Conversely, TESC overexpression reduced the abundance and functionality of CD8^+^ T cells (Figure 2, E and F). CD8^+^ T cell depletion abrogated the tumor-suppressive effect of TESC knockdown (Figure 2G and Supplemental Figure 2, A and B), establishing a direct role for CD8^+^ T cells in mediating TESC-dependent immune escape. To examine the effect of TESC on tumor antigen–specific CD8^+^ T cell responses, we implanted OVA-expressing Hepa1-6 tumors harboring either control or TESC-overexpressing constructs into C57BL/6 mice. As expected, tumors overexpressing TESC produced significantly fewer SIINFEKL–H-2Kb tetramer^+^ CD8^+^ T cells and had reduced proportions of IFN-γ^+^ and GZMB^+^ cells within the tetramer^+^ CD8^+^ T cell population compared with control tumors (Figure 2H). Consistently, IFN-γ ELISPOT analysis of tumor-infiltrating lymphocytes revealed markedly fewer IFN-γ spots in TESC-overexpressing tumors (Figure 2I). Thus, tumor TESC impaired tumor-specific CD8^+^ T cell responses in vivo.

We further investigated how intracellular TESC expression in tumor cells affects T cell activation. Although the exact mechanism remains unclear, previous studies have suggested that the immunogenicity of dying tumor cells can shape antigen-specific CD8^+^ T cell responses (10). We loaded OVA into Hepa1-6 cells and TESC-overexpressing Hepa1-6 cells and then induced apoptosis by OXA exposure to test whether TESC modulates tumor immunogenicity (Supplemental Figure 2, C and D). These apoptotic cells were then cocultured with splenocytes isolated from ovalbumin-specific T cell receptor– (OT-I)–transgenic mice, which express an OVA-specific T cell receptor (TCR) (Supplemental Figure 2C). Coculture with dead OVA-loaded control Hepa1-6 cells resulted in a dose-dependent increase in IFN-γ production, whereas coculture with dead OVA-loaded *Tesc* Hepa1-6 cells failed to elicit similar responses (Figure 2J). In addition to OT-I CD8^+^ T cells, OT-I splenocytes contain APCs, including macrophages and myeloid DCs, which are capable of processing OVA from dead tumor cells and presenting it to T cells. The markedly reduced IFN-γ production in cultures with *Tesc* tumor cells suggests that TESC may impair the capacity of APCs to cross-present antigens and activate CD8^+^ T cells. We further assessed whether TESC directly modulates T cell activation by stimulating splenic T cells from C57BL/6 mice with anti-CD3 and anti-CD28 antibodies in the presence of apoptotic control or TESC-overexpressing tumor cells. Under these conditions, the expression levels of IFN-γ, TNF-α, and GZMB were comparable between the 2 groups (Figure 2K). Consistently, the scRNA-seq analysis (GSE151530) of human HCC samples showed reduced cytotoxic T cell infiltration in tumors with high TESC expression (Figure 2L, and Supplemental Figure 2, E and F). Notably, T cells within TESC^hi^ tumors exhibited a marked downregulation of TCR signaling–associated cytotoxic programs and antigen presentation–related pathways (Figure 2M). The reduced infiltration of IFN-γ^+^ and GZMB^+^ CD8^+^ T cells in TESC^hi^ tumor tissues from patients with HCC compared with TESC^lo^ tumor tissues further corroborated the above findings (Supplemental Figure 2G). Collectively, these findings suggest that tumor-intrinsic TESC may not directly affect T cell activation but instead potentially targets APCs and reduces tumor immunity in an antigen presentation–dependent manner.

### TESC attenuates tumor immunogenicity by disrupting antigen presentation by APCs.

**Figure 3:**
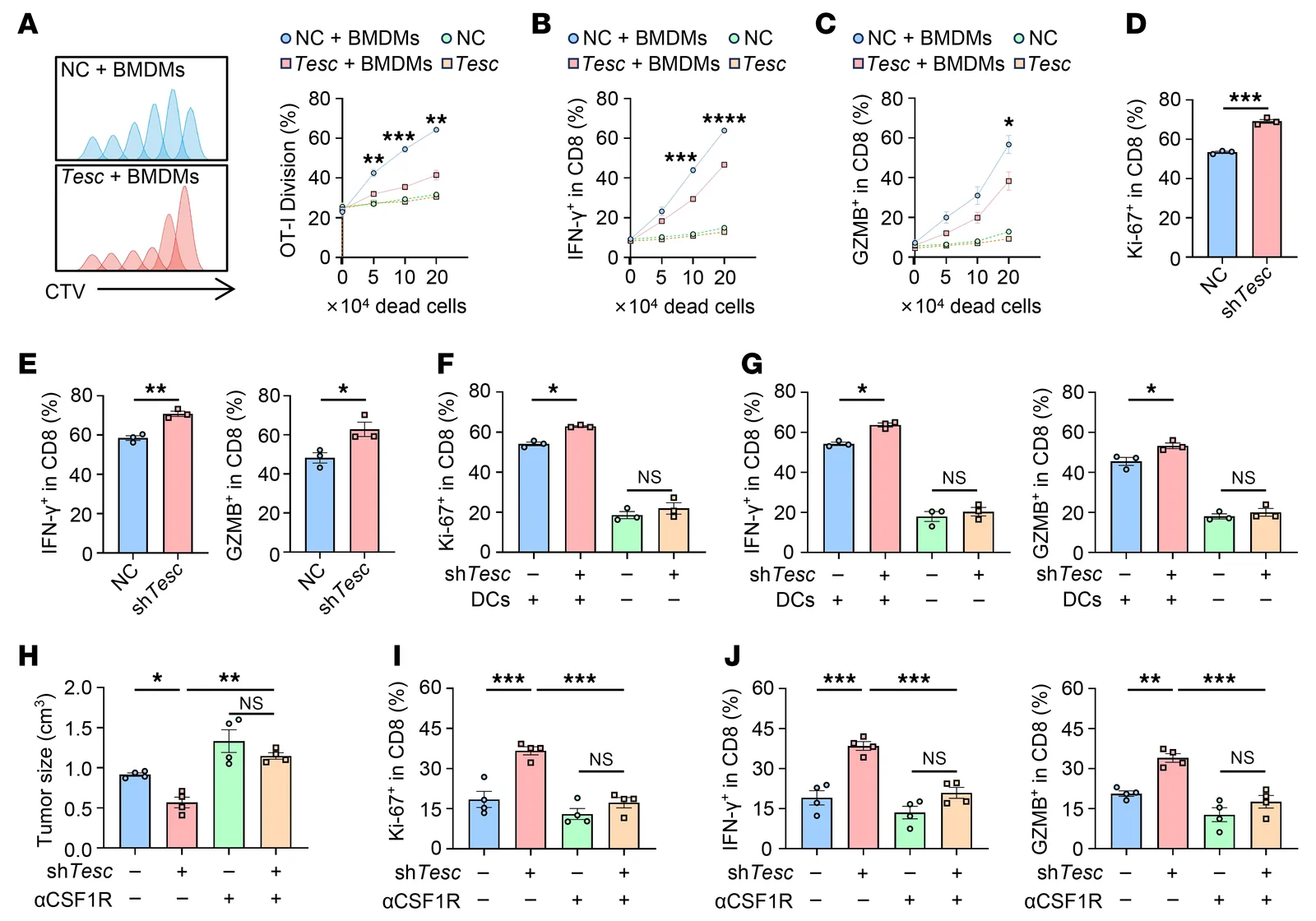
TESC attenuates tumor immunogenicity by disrupting antigen presentation by APCs. (**A**–**C**) CTV-labeled OT-I cells were cultured with different numbers of dead OVA-loaded *Tesc* Hepa1-6 cells in the presence of BMDMs for 3 days. CTV dilution (**A**) and OT-I cell function were analyzed (**B** and **C**) (*n* = 3). *P < 0.05, **P < 0.01, ***P < 0.001, and ****P < 0.0001, by 2-tailed Student’s *t* test. (**D**–**G**) OT-I cells were cultured with dead OVA-loaded sh*Tesc* H22 cells for 3 days, with macrophages (**D** and **E**) or with/without DCs (**F** and **G**). OT-I cell functions were analyzed (*n* = 3). *P < 0.05, **P < 0.01, and ***P < 0.001, by 2-tailed Student’s *t* test (**D** and **E**) and 1-way ANOVA (**F** and **G**). (**H**–**J**) Effects of macrophage depletion on tumor size (**H**) and CTL function (**I** and **J**) in sh*Tesc* H22 hepatoma–bearing mice (*n* = 4). *P < 0.05, **P < 0.01, and ***P < 0.001, by 1-way ANOVA. All data are presented as the mean ± SEM.

Damage-associated molecular patterns (DAMPs) dynamically shape the phenotype and function of APCs during tumor antigen uptake and processing, notably via macrophage- and DC-mediated phagocytosis (25–27). To investigate the potential role of tumor-intrinsic TESC in modulating APC function, we cocultured bone marrow–derived macrophages (BMDMs), CellTrace Violet–labeled (CTV-labeled) OT-I T cells, and dead OVA-expressing Hepa1-6 tumor cells either with or without TESC overexpression (Supplemental Figure 3A). We observed an increase in OT-I cell activation in a dose-dependent manner via CTV dilution in OT-I cells and an increase in the intracellular expression of IFN-γ and GZMB after coculturing with dead Hepa1-6 cells (Figure 3, A–C). However, the magnitude of OT-I cell activation was reduced following coculture with dead *Tesc* Hepa1-6 cells, suggesting that TESC impaired the capacity of dying tumor cells to trigger effective T cell activation. We performed an identical experiment with normal control (NC) and sh*Tesc* H22 cells (Figure 3, D and E). In this setting, OT-I cell activation was higher in the presence of dead sh*Tesc* H22 cells than with dead H22 cells (Figure 3, D and E). We further extended these studies from macrophages to myeloid-derived DCs. Under comparable experimental conditions, DCs were substituted for macrophages (Supplemental Figure 3, B and C). Consistently, OT-I cell activation was significantly higher in the presence of dead TESC-deficient H22 cells than with dead WT H22 cells (Figure 3, F and G). Importantly, OT-I cells failed to exhibit activation in the absence of either macrophages or DCs (Figure 3, A–C, F, and G). Thus, tumor-intrinsic TESC resulted in reduced T cell activation by targeting APCs.

We next examined the in vivo effect of tumor-derived TESC on the immune responses mediated by APCs, with a particular focus on tissue-resident macrophages. We orthotopically implanted control or sh*Tesc* H22 cells into the livers of immunocompetent mice with or without anti-CSF1R treatment to deplete macrophages (Supplemental Figure 3, D and E). Silencing TESC significantly suppressed tumor growth, but this effect was partially reversed upon macrophage depletion (Figure 3H). Consistent with these findings, CD8^+^ T cells from macrophage-depleted mice presented reduced proliferative and cytotoxic activities, as indicated by decreased Ki-67, IFN-γ, and GZMB expression (Figure 3, I and J). Collectively, these findings suggest that tumor-intrinsic TESC compromises antitumor immunity by functionally targeting both DCs and macrophages.

### TESC restrains CALR membrane translocation to inhibit macrophage function.

**Figure 4:**
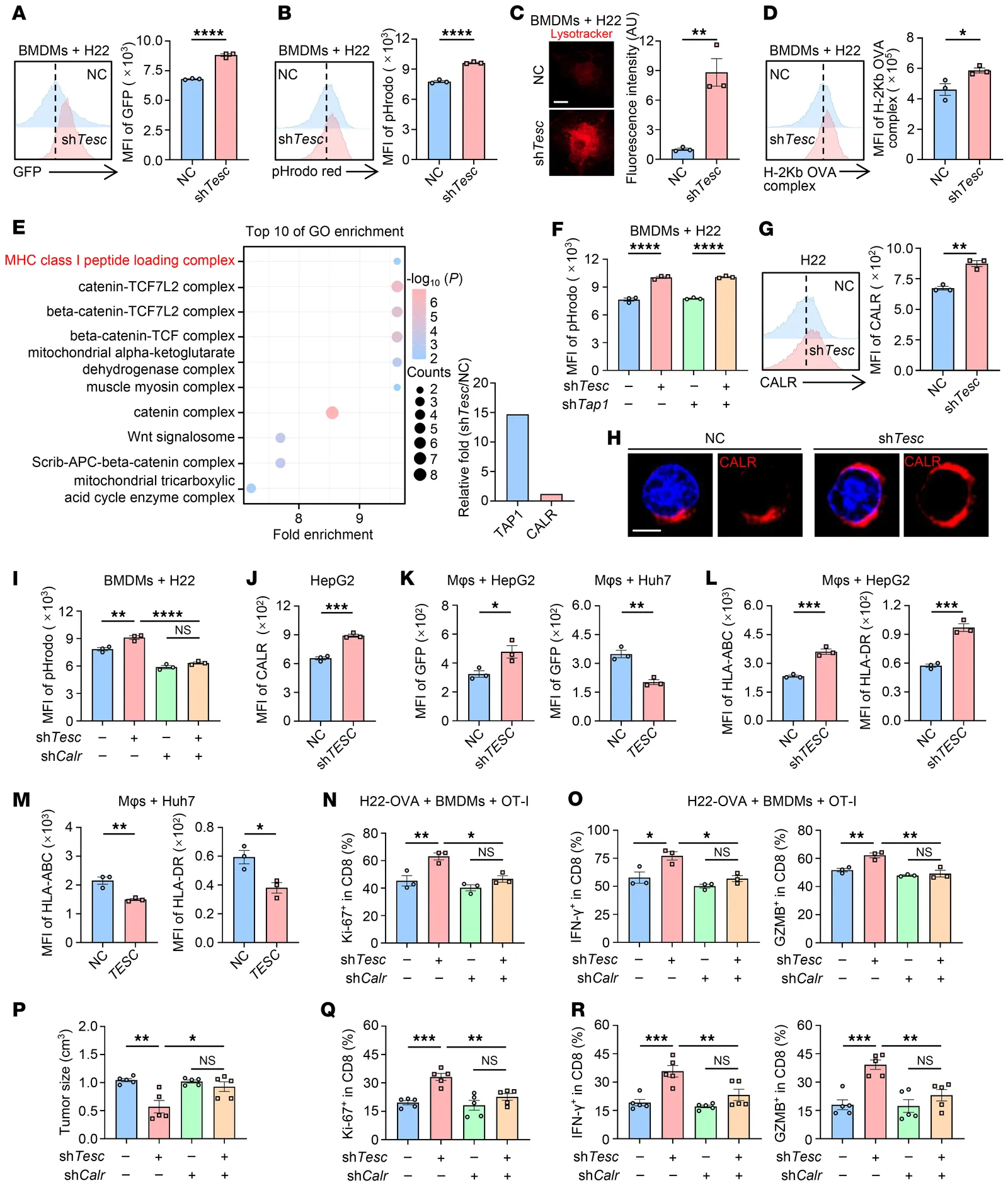
TESC restrains CALR membrane translocation to inhibit macrophage function. (**A**) FACS analysis of GFP in BMDMs cultured with dead GFP-labeled sh*Tesc* H22 cells (*n* = 3). ****P < 0.0001, by 2-tailed Student’s *t* test. (**B** and **C**) BMDMs were cultured with dead sh*Tesc* H22 cells for 20 hours, followed by pHrodo Red FACS analysis and LysoTracker confocal imaging (*n* = 3). Scale bar: 10 μm. **P < 0.01 and ****P < 0.0001, by 2-tailed Student’s *t* test. (**D**) FACS analysis of H-2Kb–OVA complexes in BMDMs cultured with dead OVA-loaded sh*Tesc* H22 cells for 48 hours (*n* = 3). *P < 0.05, by 2-tailed Student’s *t* test. (**E**) Top 10 GO biological processes enriched in sh*Tesc* H22 cells by DIA-MS proteomics, with the top pathway involving CALR and TAP1. Fisher’s exact test was used to assess significance. (**F**) FACS analysis of pHrodo Red in BMDMs cultured with dead TAP1-silenced (sh*Tap1*)-transfected sh*Tesc* H22 cells (*n* = 3). ****P < 0.0001, by 1-way ANOVA. (**G** and **H**) FACS and confocal analysis of CALR expression in sh*Tesc* H22 cells (*n* = 3). Scale bar: 10 μm. **P < 0.01, by 2-tailed Student’s *t* test. (**I**) FACS analysis of pHrodo Red in BMDMs cultured with dead CALR-silenced (sh*Calr*-transfected) sh*Tesc* H22 cells (*n* = 3). **P < 0.01 and ****P < 0.0001, by 1-way ANOVA. (**J**) FACS analysis of CALR expression in sh*TESC* HepG2 cells (*n* = 3). ***P < 0.001, by 2-tailed Student’s *t* test. (**K**–**M**) FACS analysis of GFP, HLA-ABC, and HLA-DR in macrophages (MΦs) cultured with dead GFP-labeled sh*TESC* HepG2 or *TESC* Huh7 cells (*n* = 3). *P < 0.05, **P < 0.01, and ***P < 0.001, by 2-tailed Student’s *t* test. (**N** and **O**) Functional analysis of OT-I cells cultured with dead OVA-loaded, sh*Calr*-transfected sh*Tesc* H22 cells and macrophages for 3 days (*n* = 3). *P < 0.05 and **P < 0.01, by 1-way ANOVA. (**P**–**R**) Tumor size and CTL function were analyzed after inoculation of sh*Calr*-transfected sh*Tesc* H22 cells (*n* = 5). *P < 0.05, **P < 0.01, and ***P < 0.001, by 1-way ANOVA. All data are presented as the mean ± SEM.

The phagocytosis of dead tumor cells is the initial step for APCs to capture, process, and present antigens to T cells (13, 28). To dissect the mechanism by which intrinsic TESC downregulates tumor immunogenicity, we hypothesized that tumor-derived TESC affects the nature of APC phagocytosis and, in turn, impairs APC-mediated antigen presentation and T cell activation. We tested this hypothesis by coculturing macrophages with GFP-labeled dead H22 cells or TESC-deficient H22 cells for 20 hours and assessed phagocytic uptake by quantifying intracellular GFP levels. Compared with control H22 cells, macrophages exposed to sh*Tesc* cells presented a marked increase in GFP accumulation (Figure 4A), a result also observed in vivo (Supplemental Figure 4A). This suggests that tumor-derived TESC inhibits macrophage-mediated engulfment and phagocytosis of dead tumor cells.

We next evaluated the effects of tumor-derived TESC on macrophage-mediated engulfment and phagocytosis over time (29). Similar to the above experiment (Figure 4A), we incubated macrophages with dead H22 or sh*Tesc* H22 cells for 20 hours, pulsed them with pHrodo Red–labeled particles for 20 minutes and monitored them for 40 minutes. pHrodo Red dye is pH sensitive, and its fluorescence increases in acidic phagosomes (30). We monitored red fluorescence in the macrophages and observed more beads in the phagosomes of the macrophages cultured with dead TESC-deficient cells than in those cultured with dead H22 cells (Figure 4B). We performed similar experiments with dead Hepa1-6 cells and *Tesc* Hepa1-6 cells (Supplemental Figure 4B). These data suggest that tumor-derived TESC potentially alters persistent macrophage-mediated phagocytosis. We stained macrophages with LysoTracker Deep Red, a fluorescent dye that labels acidic lysosomal compartments, to further validate this result. Confocal microscopy revealed the beads within the macrophages (Figure 4C), and the LysoTracker fluorescence intensity at the bead-containing phagosomes served as a readout for maturation. Compared with macrophages cultured with dead WT H22 cells, we detected an increase in LysoTracker fluorescence intensity at the bead areas in phagosomes in macrophages incubated with dead TESC-deficient H22 cells, suggesting induced phagosome maturation (Figure 4C). We further questioned whether the negative effect of tumor-derived TESC on macrophage phagocytosis is involved in impaired antigen presentation. We tested this hypothesis by coculturing macrophages with dead OVA-expressing H22 cells and sh*Tesc*-OVA H22 cells. Compared with those cocultured with WT H22 cells, macrophages exposed to sh*Tesc*-OVA H22 cells showed elevated levels of OVA–MHC-I complexes (Figure 4D). In contrast, macrophages incubated with TESC-overexpressing Hepa1-6 cells presented reduced OVA–MHC-I presentation (Supplemental Figure 4C), which is consistent with the findings described above, demonstrating the negative role of tumor-derived TESC in antigen-specific T cell activation (Figure 3, A–C). Consistent with the in vitro findings, TESC silencing in H22 tumors markedly increased the proportion of MHC-I^+^ and MHC-II^+^F4/80^+^ macrophages (Supplemental Figure 4D), whereas TESC overexpression in Hepa1-6 tumors decreased this population (Supplemental Figure 4E), indicating the upregulation of TESC impaired macrophage antigen-presenting capacity. In support of this observation, the scRNA-seq analysis (GSE151530) of human HCC tumors revealed that high TESC expression in malignant cells was associated with reduced phagocytosis and antigen presentation scores in tumor-associated macrophages (Supplemental Figure 4, F and G). Taken together, these results suggest that tumor-intrinsic TESC targets macrophage phagocytosis, resulting in reduced antigen presentation and T cell activation.

We next investigated how tumor-intrinsic TESC affects macrophage phagocytosis. Given that TESC had no direct effect on APC-mediated T cell activation, we hypothesized that tumor-intrinsic TESC may indirectly regulate macrophage phagocytosis through an interaction partner. To identify the potential partners of TESC at the protein level, we performed data-independent acquisition (DIA) proteomics profiling of sh*Tesc* and control H22 cells. Gene Ontology analysis revealed a significant upregulation of MHC class I peptide–loaded components, particularly transporter associated with antigen processing (TAP1), in sh*Tesc* cells (Figure 4E and Supplemental Figure 4, H and I). However, TAP1 silencing failed to alter macrophage-mediated phagocytosis or OVA–MHC-I presentation in the sh*Tesc* context (Figure 4F and Supplemental Figure 4, J and K), suggesting that TAP1 is not essential in this process. Notably, proteomics analysis revealed CALR as the most prominently altered phagocytosis-related signal upon TESC knockdown (Supplemental Figure 4L), whereas other canonical “eat-me” or “don’t-eat-me” molecules showed no significant changes, suggesting a selective effect of TESC on the CALR axis. Consistently, surface CALR exposure was markedly increased in TESC-deficient H22 cells but reduced in TESC-overexpressing Hepa1-6 cells (Figure 4, E, G, and H, and Supplemental Figure 4, H, I, and M), whereas CD47 expression levels remained comparable between sh*Tesc* H22 and TESC-overexpressing Hepa1-6 cells and their respective controls (Supplemental Figure 4, H, I, and N). Given that membrane CALR functions as an “eat-me” signal and facilitates APC phagocytosis (19, 31, 32), we hypothesized that TESC may regulate phagocytosis through the modulation of CALR membrane translocation. As expected, CALR silencing in TESC-deficient H22 cells markedly diminished phagocytic bead uptake and antigen presentation, which was comparable to the reduction observed in sh*Calr* H22 cells (Figure 4I and Supplemental Figure 4, O and P). To extend these findings to human HCC, we generated TESC-silenced HepG2 cells and TESC-overexpressing Huh7 cells (Supplemental Figure 4Q and 4R) and found that manipulation of TESC expression in both models resulted in concordant changes in CALR surface exposure: TESC silencing in HepG2 significantly increased CALR membrane localization, whereas TESC overexpression in Huh7 markedly reduced CALR surface exposure (Figure 4J and Supplemental Figure 4S). Consistently, macrophages and DCs differentiated from human peripheral blood monocytes exhibited increased phagocytosis of GFP-labeled TESC-silenced HepG2 cells, accompanied by elevated HLA-ABC and HLA-DR expression, whereas the opposite effect was observed in the coculture system with TESC-overexpressing Huh7 cells (Figure 4, K–M, and Supplemental Figure 4, T–V).

To determine the functional consequence of CALR in CD8^+^ T cell immunity, we cultured OT-I cells with macrophages in the presence of OVA-loaded dead tumor cells. TESC-deficient H22 cells induced robust OT-I proliferation and activation, as evidenced by increased Ki-67, IFN-γ, and GZMB expression, whereas CALR silencing abolished these effects (Figure 4, N and O). Similarly, in vivo, CD8^+^ T cell–mediated cytotoxicity toward TESC-deficient hepatomas was abrogated by concomitant CALR silencing, leading to tumor growth rates comparable to those of controls (Figure 4, P–R). Moreover, RNA-seq of macrophages that had engulfed TESC-overexpressing versus control tumor cells (Genome Sequence Archive [GSA]: CRA037701) revealed that broad suppression of gene programs related to actin cytoskeletal dynamics, ion channel activity, APC costimulation and maturation (CD40, CD86, MHC class I and II), and cytokine production, including IL-6 (Supplemental Figure 4W). Collectively, these results demonstrate that tumor-intrinsic TESC restrains CALR membrane translocation, thereby suppressing macrophage phagocytosis and antigen presentation and dampening CD8^+^ T cell–mediated antitumor immunity

### TESC regulates CALR surface exposure by modulating calcium homeostasis and attenuating ER stress.

**Figure 5:**
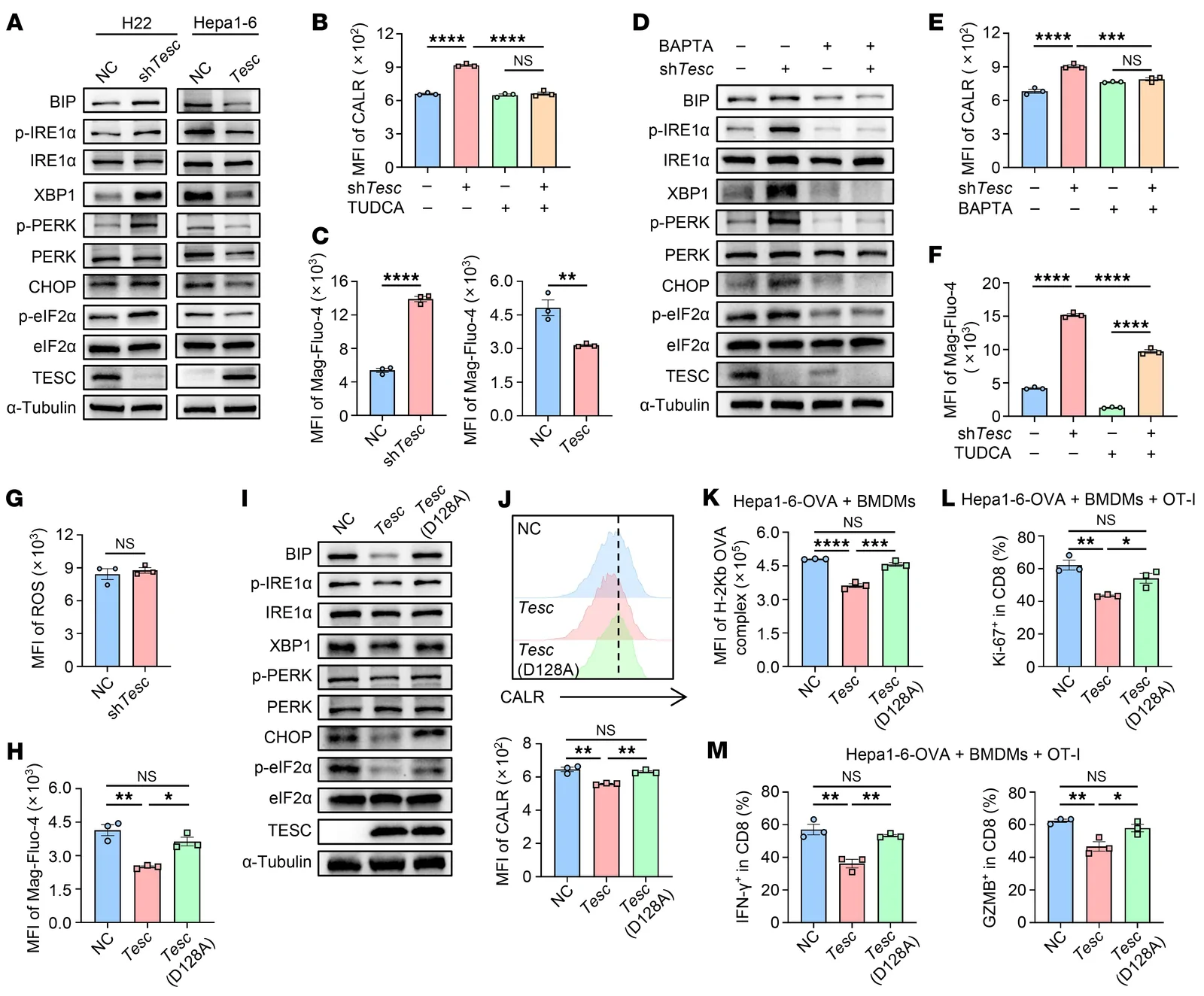
TESC regulates CALR surface exposure by modulating calcium homeostasis and attenuating ER stress. (**A**) Immunoblot analysis of ER stress signaling activation in sh*Tesc* H22 or *Tesc* Hepa1-6 cells. (**B**) FACS analysis of CALR expression in sh*Tesc* H22 cells treated with TUDCA for 24 hours (*n* = 3). ****P < 0.0001, by 1-way ANOVA. (**C**) FACS analysis of ER calcium level in sh*Tesc* H22 or *Tesc* Hepa1-6 cells stained with Mag–Fluo-4–AM (*n* = 3). **P < 0.01 and ****P < 0.0001, by 2-tailed Student’s *t* test. (**D**) Immunoblot analysis of ER stress signaling activation in sh*Tesc* H22 cells left untreated or incubated with BAPTA for 1 hour. (**E**) FACS analysis of CALR expression in sh*Tesc* H22 cells treated with BAPTA for 1 hour (*n* = 3). ***P < 0.001 and ****P < 0.0001, by 1way ANOVA. (**F**) FACS analysis of ER calcium levels in sh*Tesc* H22 cells left untreated or incubated with TUDCA for 24 hours (*n* = 3). ****P < 0.0001,by 1-way ANOVA. (**G**) FACS analysis of ROS level in sh*Tesc* H22 cells (*n* = 3). Significance was determined by 2-tailed Student’s *t* test. (**H**) FACS analysis of ER calcium level in *Tesc-* or D128A point mutant TESC-overexpressing [*Tesc* (D128A)] Hepa1-6 cells (*n* = 3). *P < 0.05 and **P < 0.01, by 1-way ANOVA. (**I**) Immunoblot analysis of ER stress signaling activation in *Tesc* or *Tesc* (D128A) Hepa1-6 cells. (**J**) FACS analysis of CALR expression in *Tesc* or *Tesc* (D128A) Hepa1-6 cells (*n* = 3). **P < 0.01, by 1-way ANOVA. (**K**) FACS analysis of H-2Kb–OVA complexes in BMDMs cultured with dead OVA-loaded *Tesc* or *Tesc* (D128A) Hepa1-6 cells for 48 hours (*n* = 3). ***P < 0.001 and ****P < 0.0001, by 1-way ANOVA. (**L** and **M**) Functional analysis of OT-I cells cultured with dead OVA-loaded *Tesc* or *Tesc* (D128A) Hepa1-6 cells and macrophages for 3 days (*n* = 3). *P < 0.05 and **P < 0.01, by 1-way ANOVA. All data are presented as the mean ± SEM.

Having established the importance of TESC in modulating CALR surface exposure and thereby governing macrophage-mediated phagocytosis and antigen presentation, we next sought to elucidate the underlying molecular mechanism. CALR is a key ER-resident chaperone that is essential for maintaining protein-folding homeostasis (33). Upon ER stress activation, CALR can be aberrantly translocated to the cell surface via the unfolded protein response (UPR), where it acts as a canonical “eat-me” signal to facilitate immune recognition and phagocytosis (33, 34). To determine whether TESC influences CALR membrane exposure by regulating ER stress, we first examined UPR pathway activation in cells with different levels of TESC expression. Immunoblot analyses revealed that TESC silencing robustly activated UPR signaling, whereas TESC overexpression markedly attenuated ER stress (Figure 5A). Treatment of sh*Tesc* and NC H22 cells with the ER stress inhibitor TUDCA abolished the increase in CALR surface exposure induced by TESC depletion (Figure 5B), indicating that TESC limits CALR membrane translocation through the suppression of ER stress. TESC contains a canonical EF-hand domain capable of binding cytosolic Ca^2+^ (35). Given the central role of calcium signaling in UPR activation and ER homeostasis (36, 37), we hypothesized that TESC buffers excess cytosolic Ca^2+^, thereby preventing ER Ca^2+^ overload and subsequent stress responses. We tested this hypothesis using the ER-specific calcium probe Mag–Fluo-4–AM to quantify ER luminal Ca^2+^ levels. Notably, TESC deficiency increased ER Ca^2+^ accumulation, whereas TESC overexpression reduced ER Ca^2+^ levels (Figure 5C and Supplemental Figure 5, A and B), suggesting that TESC restricts Ca^2+^ influx into the ER to maintain homeostasis. We next asked whether cytosolic calcium is required for the ability of TESC to regulate macrophage phagocytosis and antigen presentation. The chelation of cytosolic Ca^2+^ with BAPTA significantly alleviated ER stress and CALR membrane exposure in TESC-deficient cells (Figure 5, D and E). Importantly, the direct alleviation of ER stress did not significantly alter ER Ca^2+^ levels (Figure 5F and Supplemental Figure 5C), indicating that Ca^2+^ overload preceded UPR activation, not vice versa. These findings support a model in which TESC regulates CALR membrane translocation by maintaining cytosol-to-ER calcium homeostasis.

Calcium overload may also perturb mitochondrial homeostasis, leading to elevated mitochondrial Ca^2+^ and ROS production, which secondarily activate the UPR (37–39). We further assessed mitochondrial Ca^2+^ levels using Rhod-2–AM and intracellular ROS levels in cells with different TESC expression levels to exclude this alternative pathway. Neither parameter changed significantly (Figure 5G and Supplemental Figure 5, A and B), suggesting that TESC regulates the UPR predominantly through the cytosol/ER calcium axis rather than via mitochondrial dysfunction. To further determine whether the calcium-binding ability of TESC is essential for its function, we generated a point mutation (D128A) in the EF-hand domain known to disrupt calcium coordination (35). This mutant failed to suppress ER Ca^2+^ accumulation (Figure 5H), reinstated UPR activation (Figure 5I), and restored CALR membrane translocation (Figure 5J), despite its overexpression. Consistent with our previous results (Figure 3, A–C, and Supplemental Figure 4C), macrophages exposed to TESC-overexpressing cells had significantly greater suppressed antigen presentation and T cell activation than did Hepa1-6 cells, whereas the D128A mutation abolished these effects (Figure 5, K–M). Thus, TESC restricts ER stress–induced CALR exposure by buffering cytosolic Ca^2+^, thereby suppressing macrophage phagocytosis and antitumor immunity in HCC.

### Targeting H3K4me3-induced TESC overcomes immune suppression and potentiates the efficacy of PD-1 combination therapy in HCC.

**Figure 6:**
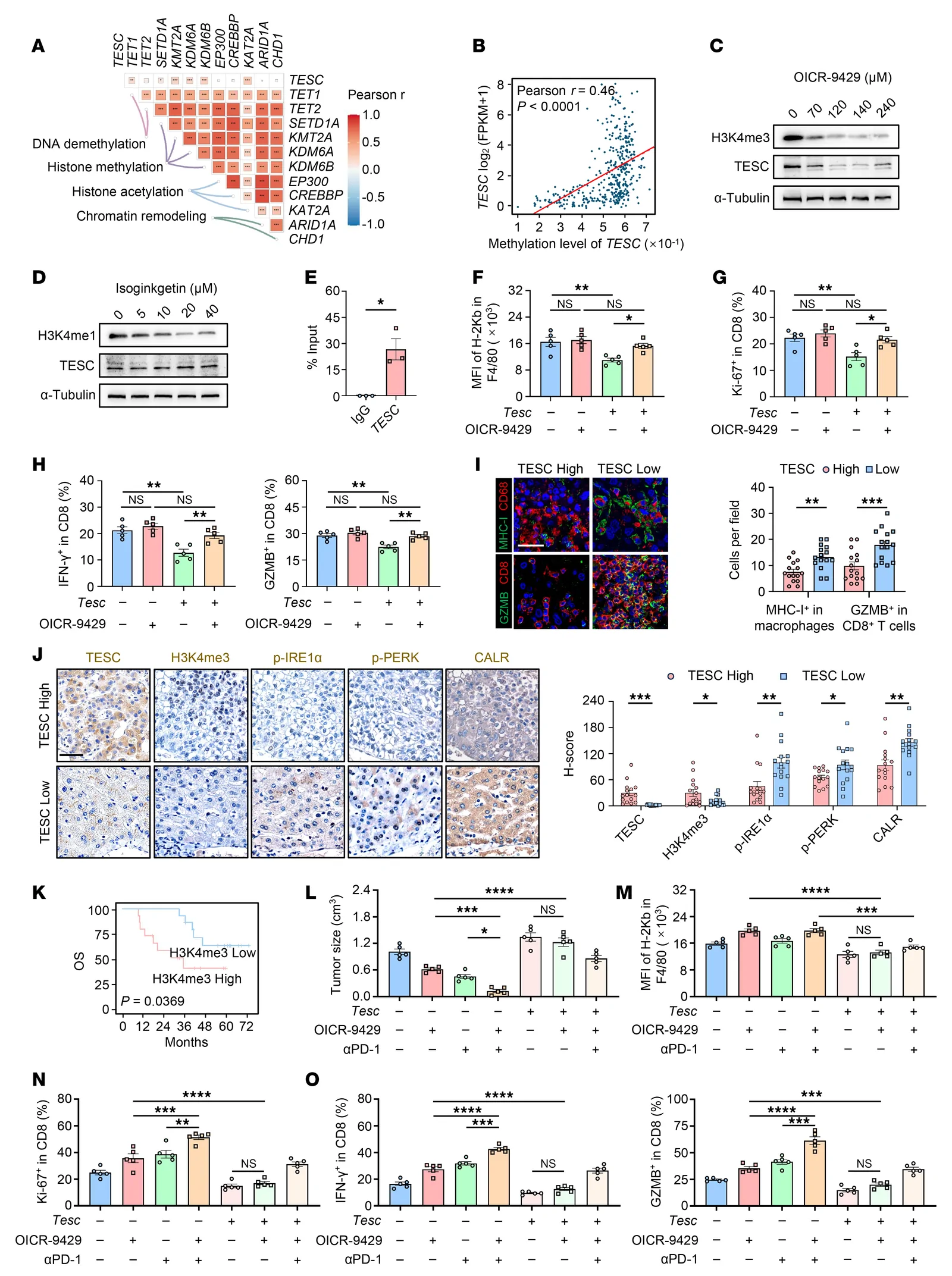
Targeting H3K4me3-induced TESC overcomes immune suppression and potentiates the efficacy of PD-1 combination therapy in HCC. (**A**) Heatmap of correlations between TESC expression and epigenetic regulator–related genes (TCGA-HCC). (**B**) Scatter plot from the MethMarkerDB database showing the relationship between TESC methylation and expression. Pearson’s correlation coefficient was applied. (**C** and **D**) Immunoblot analysis of TESC and H3K4me3 or H3K4me1 expression in H22 cells treated with OICR-9429 or isoginkgetin for 24 hours. (**E**) ChIP-qPCR showing the binding of H3K4me3 to TESC promoter in H22 cells (*n* = 3). *P < 0.05, by 2-tailed Student’s *t* test. (**F**–**H**) *Tesc* Hepa1-6 hepatoma–bearing mice were untreated or injected with OICR-9429 (*n* = 5). H-2Kb levels in macrophages (**F**) and CTL function (**G** and **H**) were analyzed. **P < 0.01, by 1-way ANOVA. (**I** and **J**) Cohort 3 HCC patients were grouped by median TESC expression. Tumor TESC, H3K4me3, phosphorylated IRE1α (p-IRE1α), p-PERK, and CALR levels were analyzed by IHC. MHC-I^+^ macrophages and GZMB^+^ CTLs were analyzed by immunofluorescence. Scale bars: 50 μm. *P < 0.05, **P < 0.01, and ***P < 0.001, by 2-tailed Student’s *t* test. (**K**) Correlation of H3K4me3 levels with OS of cohort 3 HCC patients grouped by median H3K4me3 expression. (**L**–**O**) *Tesc* H22 hepatoma–bearing mice were untreated or injected with OICR-9429, anti–PD-1 antibody, or their combination (*n* = 5). Tumor size (**L**), H-2Kb levels in macrophages (**M**), and CTL function (**N** and **O**) were analyzed. *P < 0.05, **P < 0.01, ***P < 0.001, and ****P < 0.0001, by 1-way ANOVA. All data are presented as the mean ± SEM.

Epigenetic modifications, including DNA methylation, histone modifications, and chromatin remodeling, are critical regulators of gene regulation and cellular plasticity (40). To explore whether TESC expression is epigenetically regulated, we analyzed HCC epigenetic alterations from TCGA dataset and observed a strong positive correlation between TESC expression and the expression of multiple histone methyltransferases (Figure 6A), suggesting a role for histone methylation in the control of TESC transcription. Consistently, an analysis of the MethMarkerDB database revealed a positive association between TESC expression and promoter DNA methylation (Figure 6B). ChIP sequencing data from normal human liver tissues (ENCSR000FYQ) showed marked enrichment of H3K4me1 and H3K4me3 at the TESC promoter region (Supplemental Figure 6A). Targeted inhibition of H3K4me3 using the selective antagonist OICR-9429 markedly suppressed TESC expression in both H22 and HepG2 cells in a dose-dependent manner (Figure 6C and Supplemental Figure 6B), whereas pharmacologic blockade of H3K4me1 had no effect (Figure 6D and Supplemental Figure 6C). Consistently, ChIP–quantitative PCR (qPCR) analysis confirmed robust H3K4me3 occupancy at the TESC promoter, supporting a direct role for H3K4me3 in driving TESC transcription (Figure 6E). Notably, TESC knockdown and overexpression did not alter global H3K4me3 levels (Supplemental Figure 6D), suggesting that H3K4me3 acted upstream of TESC and not vice versa. Similarly, in mice bearing Hepa1-6 hepatoma, injection of OICR-9429 effectively reduced tumor TESC expression (Supplemental Figure 6, E and F). Importantly, OICR-9429 treatment restored antigen presentation by tumor-associated macrophages and rescued CD8^+^ T cell effector responses previously suppressed by TESC (Figure 6, F–H), indicating that pharmacological targeting of TESC can reinvigorate antitumor immunity. Notably, in HCC tissues derived from patients receiving anti–PD-1 therapy, low TESC expression was associated with reduced H3K4me3 levels, increased ER stress, increased CALR surface exposure, enhanced macrophage antigen presentation, and increased CD8^+^ T cell cytotoxic activity (Figure 6, I and J). Accordingly, intratumoral H3K4me3 expression showed an acceptable ability to predict the response to immunotherapy (AUC = 0.75, *P* < 0.05), and reduced intratumoral H3K4me3 levels in patients with HCC significantly correlated with improved OS after immunotherapy (Figure 6K and Supplemental Figure 6G), suggesting that this epigenetic axis is a key determinant of immune responsiveness.

Since H3K4me3 drives transcription and impairs immune activation, we next tested whether targeting this epigenetic axis could increase the efficacy of PD-1 blockade in tumors. In an orthotopic *Tesc* H22 hepatoma model, treatment with the selective H3K4me3 inhibitor OICR-9429, the anti–PD-1 antibody, or their combination led to the suppression of TESC expression and tumor growth (Figure 6L and Supplemental Figure 6H), with the greatest therapeutic benefit observed in the combination group (Figure 6L) The combination of OICR-9429 and anti–PD-1 antibody was found to be safe (Supplemental Figure 6I). This response was associated with enhanced macrophage-mediated antigen presentation and improved CD8^+^ T cell effector function, as indicated by increased expression of H-2Kb, Ki-67, IFN-γ, and GZMB (Figure 6, M–O). Notably, these immune enhancements and antitumor effects were abolished in TESC-overexpressing tumors, confirming that the therapeutic efficacy of OICR-9429 depends on effective TESC inhibition (Figure 6, M–O). In a sg*P53*/*Myc*-driven spontaneous hepatoma model characterized by intrinsic resistance to ICB, enforced TESC overexpression further exacerbated resistance to both anti–PD-1 monotherapy and combined OICR-9429 and anti–PD-1 treatment, accompanied by reduced macrophage antigen-presenting capacity and impaired CTL function (Supplemental Figure 6, J–O). Moreover, in an acquired anti–PD-1–resistant orthotopic hepatoma model established through iterative cycles of anti–PD-1 treatment followed by tumor re-isolation and re-implantation (Supplemental Figure 6P), TESC expression was markedly elevated in the resistant tumor (Supplemental Figure 6Q), and pharmacological inhibition of TESC using OICR-9429 significantly restored sensitivity to anti–PD-1 therapy (Supplemental Figure 6R). Thus, H3K4me3-driven TESC functions as an epigenetic barrier to antitumor immunity in HCC and represents a target for combination therapy.

## Discussion

ICB has revolutionized cancer therapy, yet its clinical efficacy in HCC remains limited due to the intrinsic resistance mechanisms embedded within the immunosuppressive tumor microenvironment (TME) (4, 41–43). Here, we identified TESC, a calcium-binding EF-hand protein, as a previously unrecognized tumor-intrinsic modulator and phagocytic checkpoint that promotes immune evasion and compromises the efficacy of ICB in HCC. We used multiple complementary strategies to map the regulation, pathogenic influences, and clinical relevance of this molecule to cancer progression and ICB therapy. These findings highlight a pivotal role for tumor-intrinsic molecules in shaping the immune characteristics of HCC and provide insights into the mechanism underlying immunotherapy resistance.

Studies have revealed that TESC, a calcium-binding EF-hand protein, may be involved in cellular stress, metabolism, proliferation, and tumorigenesis (44). Several studies have detected elevated levels of TESC in cancers, such as thyroid cancer, colorectal cancer, and cholangiocarcinoma (45–47). Unexpectedly, we found that TESC is an immunoregulatory molecule in the context of tumor immunity. We functionally validated the relationship between TESC and tumor immunity in multiple murine hepatoma models. Hence, our work revealed that tumor-derived TESC functions as a previously unappreciated intrinsic resistance mechanism in tumor immunity and immunotherapy.

Although TESC exerted potent immunosuppressive effects on various hepatoma-bearing models in vivo and its high expression was associated with a poor clinical response to ICB, the precise mechanisms by which TESC disrupts antitumor immunity remain unclear. We observed that TESC was markedly overexpressed in HCC cells, and its expression correlated inversely with tumor sensitivity to ICB. Given that TESC is a nonsecretory protein, we hypothesized that TESC, similar to MYC or NLRC5 (48, 49), might interfere with MHC-I antigen presentation and directly facilitate tumor immune escape by evading T cell recognition. Unfortunately, TESC expression in tumor cells did not directly inhibit T cell activation under either antigen-specific or polyclonal TCR stimulation. Intriguingly, when T cells were cocultured with APCs in the presence of either TESC-overexpressing or TESC-deficient apoptotic tumor cells, we found that T cell activation was substantially impaired in the former group but not in the latter group. These results prompted us to speculate that tumor-derived TESC may modulate APC function and, in turn, regulate APC-mediated T cell priming.

We initially examined the potential role of tumor cell–intrinsic TESC in APC-mediated phagocytosis to explore this possibility. Phagocytosis is an early step during APC-mediated antigen capture, processing, and presentation (13). Indeed, we have shown that tumor cell–derived TESC negatively affected APC-mediated phagocytosis, accompanied by reduced antigen presentation. Since no prior studies have reported the regulatory role of TESC in APC function, and given its nature as a Ca^2+^ sensor (35), we speculated that TESC may modulate phagocytic activity by altering intracellular calcium–dependent signaling. Consistent with this possibility, our proteomics profile revealed close interactions between TESC and key components of the MHC class I peptide–loading complex, notably TAP1 and CALR. Strikingly, silencing CALR, but not TAP1, abolished the enhanced phagocytosis and antigen presentation observed in macrophages exposed to TESC-deficient tumor cells. Mechanistically, the loss of TESC increased cytosolic Ca^2+^ levels, induced ER Ca^2+^ overload and stress, and promoted CALR exposure on the tumor cell surface — an established prophagocytic “eat-me” signal (19). These findings suggest that TESC attenuates ER stress by modulating cytosolic calcium levels, thereby reducing CALR membrane translocation and suppressing CALR-mediated APC phagocytic activity. Consistent with prior studies showing that surface CALR is essential for immunogenic cell clearance (19, 50), particularly when counteracting “don’t-eat-me” signals such as CD47 or CD24, we propose that TESC functions as an intracellular checkpoint that suppresses CALR-mediated phagocytosis. Indeed, in patients with HCC treated with ICB, low TESC expression correlated with elevated ER stress, increased membrane CALR expression, and increased APC-driven CD8^+^ T cell activation. As CALR exposure is a prerequisite for immunogenic tumor cell death induced by chemotherapeutic drugs such as OXA and mitoxantrone (19), our findings extend this paradigm by positioning TESC as a key negative regulator of CALR trafficking. In addition to recent advances in identifying CD47–SIRPα, CD24–Siglec-10, and Siglec-15 as phagocytosis checkpoints (16, 51, 52), our study introduces TESC as an intracellular modulator of the phagocytic synapse. Together, these findings suggest that TESC represents a previously unrecognized phagocytosis checkpoint that governs the immunogenicity of tumor cells by restricting CALR surface exposure and macrophage-mediated clearance. Therefore, a better understanding of the regulatory network of TESC in cancer cells in the tumor environment would be helpful for developing rational macrophage-based designs for anticancer therapies that can amplify the antitumorigenic function of ICB.

Epigenetic dysregulation is a hallmark of cancer (53). While previous studies have shown that genetic and epigenetic alterations can impair the IFN-γ/MHC-I axis and lead to T cell dysfunction and immune escape (11), whether these tumor-intrinsic changes also affect APC-mediated phagocytosis, thereby contributing to ICB resistance, remains unclear. In the present study, through integrated multiomics analyses, we identify a strong positive association between TESC expression and multiple histone methyltransferases in HCC, with H3K4me3 emerging as a key epigenetic driver of TESC upregulation. Selective inhibition of H3K4me3 using OICR-9429 consistently suppressed TESC expression in both transplanted and spontaneous murine hepatoma, restored CALR surface exposure, reactivated the phagocytic function of macrophages, and increased the efficacy of ICB. Notably, in an acquired anti–PD-1–resistant orthotopic hepatoma model through iterative anti–PD-1 treatment and tumor re-implantation, pharmacological inhibition of TESC similarly reinstated sensitivity to anti–PD-1 therapy. These results suggest that TESC represents a therapeutically actionable epigenetic-immune node that mechanistically links tumor-intrinsic chromatin remodeling to impaired phagocytic surveillance and immune evasion.

In addition to its biological importance, our work may inform the clinical management of patients with HCC receiving anti–PD-1 (L1) therapy, suggesting that combination with epigenetic inhibitors of TESC, such as OICR-9429, could enhance therapeutic efficacy. Although our work establishes a tumor-intrinsic role for TESC in restraining CALR-dependent phagocytosis and immune surveillance, the generalizability of these findings across diverse HCC etiologies, distinct TMEs, or other cancer types remains to be determined. Additionally, our models primarily use murine transplanted and spontaneous hepatomas, which may not fully recapitulate human tumor heterogeneity. Future studies should evaluate the clinical translation of pharmacological TESC inhibition (e.g., OICR-9429), including therapeutic feasibility, safety, and optimal dosing in humans. Collectively, these directions highlight the potential of targeting the H3K4me3/TESC axis to improve ICB outcomes and guide rational combination strategies for HCC.

## Methods

### Sex as a biological variable.

Our study examined male and female animals, and similar findings are reported for both sexes.

### Patients and specimens.

Tumor samples were collected from 64 patients with pathologically confirmed HCC at the Sun Yat-sen University Cancer Center. Samples from patients with concurrent autoimmune disease, HIV, or syphilis were excluded. Tumor biopsy samples from 14 patients with HCC collected prior to OXA-based chemotherapy plus ICB (anti–PD-1 antibody) treatment between May 2019 and June 2020 were used for RNA-seq (cohort 1). Patients in cohort 1 were enrolled from a clinical trial (NCT03869034). Among the 29 evaluable patients, 14 (48.3%) responded to treatment, whereas 15 (51.7%) did not respond. Tumor samples from 20 patients with HCC who exclusively underwent surgical resection between July 2017 and September 2017 were subjected to immunofluorescence. Tumor samples from 30 patients with comprehensive follow-up data who underwent surgical resection after receiving OXA-based chemotherapy plus ICB treatment between April 2019 and July 2022 were used for both immunofluorescence and IHC analyses (cohort 3). Tumor biopsy specimens from 20 patients with comprehensive follow-up data who received OXA-based chemotherapy between July 2020 and June 2022 were used for IHC analysis (cohort 4). Detailed patient information is provided in Supplemental Table 1.

### Animal studies.

WT C57BL/6 mice, BALB/C mice, nude mice, and OT-I–transgenic mice, all aged 5–8 weeks, were purchased from Beijing Vital River Laboratory Animals.

For the orthotopic hepatoma models, Hepa1-6 cells and H22 cells were inoculated into C57BL/6, BALB/C, and nude mice, respectively. A total of 1 × 10^6^ hepatoma cells were suspended in 20 μL of a mixture of PBS and Matrigel at a 1:1 volume ratio and then injected into the subcapsular region of the liver. Tumor size (tumor length × width × height/2) was measured for a comparative analysis. For the spontaneous hepatoma models, a plasmid mixture containing 10 μg sg*P53*, 12 μg *Myc*, 20 μg NC/sg*Tesc* or NC/*Tesc*, and SB100 transposase plasmid at 1/4 of the total plasmid mass was diluted in 0.9% saline to a final volume corresponding to 10% of the mouse body weight and hydrodynamically injected into mice via the tail vein within 5–7 seconds. The maximal diameter of macroscopic tumor nodules was analyzed. For the anti–PD-1–resistant mouse model, mice bearing Hepa1-6 hepatomas were treated with anti–PD-1 or IgG in each cycle, and tumors with the highest burden were harvested for ex vivo tumor cell isolation and expansion, followed by re-implantation into mice. After 4 sequential cycles, anti–PD-1–resistant and anti–PD-1–sensitive mouse models were established. The remaining detailed procedures of the animal experiments are shown in Figure 1, H–J, M, and O, Supplemental Figure 2A, Supplemental Figure 3D, and Supplemental Figure 6, E, J and P.

### Cell lines and cell culture.

The murine hepatoma cell lines Hepa1-6 and Hepa1c1c7, the human hepatoma cell lines HepG2, SNU449, LM3, and Hep3B, and HEK293T were obtained from the American Type Culture Collection (ATCC). The murine hepatoma cell line H22 and the human hepatoma cell line Huh7 were obtained from Procell. These cell lines were cultured in DMEM or RPMI 1640 supplemented with 10% FBS and 1% penicillin-streptomycin in cell culture dishes. All the cell lines were routinely authenticated using short tandem repeat analysis and tested for mycoplasma contamination using single-step PCR.

### Flow cytometry (FACS).

Single-cell suspensions were prepared from fresh tumor tissue as previously described (41). Briefly, tumors were cut into 2–3 mm^3^ pieces and dissociated into single cells using a mouse tumor dissociation kit for 25 minutes at 37°C. The resulting cell suspensions were filtered through a 70 μm strainer and resuspended in PBS containing 2% BSA. RBCs were lysed with RBC lysis buffer. For surface staining, the samples were incubated with a cocktail of antibodies in cell-staining buffer for 30 minutes at 4°C. For intracellular staining, the samples were first stimulated for 6 hours at 37°C with a cell stimulation cocktail, followed by treatment with a fixation/permeabilization kit and then incubation with antibodies. For intranuclear staining, the samples were treated with the Pharmingen Transcription Factor Buffer Set (BD Biosciences, Catalog 562574) according to the manufacturer’s instructions and incubated with antibodies. Finally, the cells were washed, resuspended in PBS at 4°C, and analyzed using a flow cytometer (CytoFLEX). The data were processed using FlowJo software.

For the apoptosis assay, H22 cells were treated with 120 μM OXA, and Hepa1-6, HepG2, and Huh7 cells were treated with 700 μM OXA for 48 hours. The tumor cells were stained using the Annexin V-AF647/PI Apoptosis Detection Kit (GOONIE, Catalog 100-102) according to the manufacturer’s protocol and analyzed using a flow cytometer (CytoFLEX). The data were processed using FlowJo software.

### IHC analysis.

Paraffin-embedded tumor samples were cut into 4 μm thick slices. After the sections were baked for 1 hour at 65°C, the paraffin was removed by dewaxing in xylene, followed by rehydration through a graded ethanol series. Antigen retrieval was performed using EDTA buffer. The sections were then incubated with endogenous peroxidase–blocking buffer for 15 minutes to inhibit endogenous peroxidase activity and subsequently treated with blocking buffer for 30 minutes. The samples were incubated with primary antibodies overnight at 4°C. After washes with TBST, the samples were incubated with an HRP-conjugated secondary antibody for 30 minutes at 37°C. Signals were visualized using DAB substrate, and the reaction was terminated by rinses with distilled water. The sections were dehydrated through a graded ethanol series and mounted with neutral balsam before being counterstained with hematoxylin. The slides were scanned using a KONFOONG scanner (KONFOONG Bioinformation).

### Immunofluorescence staining.

Immunofluorescence staining was performed using a PANO 7-plex IHC kit. The procedures, from paraffin-embedded tissue section hydration to the secondary antibody incubation, were performed according to the protocol outlined in *IHC* *analysis*. Then, the sections were incubated with fluorescent dyes and diluted in the tyramine working solution for 10 minutes at room temperature. Microwave-based antigen retrieval was performed in EDTA buffer (pH 9.0). The sections were incubated with blocking buffer for 30 minutes at room temperature. The staining cycle procedure was repeated for each primary antibody until the desired number of cycles was completed. The sections were mounted with anti-fluorescence quenching sealing solution containing DAPI. The slides were scanned using the KONFOONG scanner.

For immunofluorescence staining of surface CALR in hepatoma cells, cells grown on confocal dishes were fixed with 4% paraformaldehyde for 15 minutes at room temperature. After 3 washes with PBS, the cells were blocked with 5% BSA without permeabilization. The cells were then stained with a primary anti-CALR antibody, followed by an incubation with a secondary antibody conjugated to Alexa Fluor 488.

### Immunoblotting.

The processed cells were washed 3 times with PBS, resuspended in lysis buffer, and incubated on ice for 30 minutes. Following ultrasonic disruption and centrifugation at 10,000*g* for 15 minutes, the supernatants were collected, mixed with loading buffer, and heated at 95°C for 10 minutes. The total protein solutions were loaded onto a SDS-PAGE gel and then electrotransferred to PVDF membranes. After blocking with 5% BSA, the membranes containing the target proteins were incubated with specific antibodies overnight at 4°C. Finally, the target protein was detected using specific antibodies and a commercial ECL kit.

### Cell proliferation assay.

Hepa1-6 or H22 hepatoma cells were plated at a density of 2 × 10^3^ cells per well in a 96-well cell culture plate. The cell density was measured every 24 hours using the Cell Counting Kit-8 (CCK-8) (MCE, catalog HY-K0301) reagent according to the manufacturer’s protocol, and the cells were incubated for 1 hour at 37°C. The OD value was assessed at 450 nm.

### ChIP assay.

ChIP assays were conducted to investigate the association between H3K4me3 and the TESC promoter. The assays were performed using an EZ-Magna ChIP kit (Sigma-Aldrich, catalog 17-10086) according to the manufacturer’s instructions. After the crosslinks were reversed, the DNA immunoprecipitated with the H3K4me3 antibody was analyzed by qRT–PCR.

### ELISA.

For the analysis of splenocyte activation, splenocytes from OT-I TCR–transgenic mice were activated and incubated with dead OVA-loaded tumor cells at a 2:1 ratio for 3 days. IFN-γ concentrations in the supernatants from the incubation of splenocytes with dead tumor cells were measured using ELISA kits (MultiSciences, catalog 70-EK280/3) according to the manufacturer’s instructions.

### ER and mitochondrial calcium measurements.

Calcium levels in the ER and mitochondria were monitored using the specific calcium-sensitive fluorescence indicators Mag-Fluo-4-AM and Rhod2-AM, respectively. After washes with HBSS, tumor cells were incubated with 2 μM Mag–Fluo-4–AM or 5 μM Rhod2-AM for 40 minutes at 37°C in the dark. The fluorescence intensity was measured to assess calcium levels via fluorescence microscopy or FACS.

### Measurement of ROS levels.

Intracellular ROS levels were measured using the ROS-sensitive fluorescence indicator H2DCFDA. After washes with PBS, the tumor cells were incubated with 5 μM H2DCFDA for 30 minutes at 37°C in the dark. ROS levels were quantified by measuring the fluorescence intensity using FACS.

### Detection of cell membrane CALR levels.

Tumor cells were collected, washed 3 times with PBS, and then resuspended in cell-staining buffer. The cells were incubated with a CALR antibody at 4°C for 30 minutes. Next, the cells were washed 3 times with PBS and incubated with a secondary antibody conjugated to Alexa Fluor 488 at 37°C for 30 minutes. Finally, the cells were washed, resuspended in PBS at 4°C, and analyzed using a flow cytometer.

### Mouse CD8^+^ T cell isolation and activation.

CD8^+^ T cells or OT-I cells were isolated from the spleens of WT mice or OT-I TCR–transgenic mice. CD8^+^ T cells or OT-I cells were obtained using the EasySep Mouse CD8^+^ T cell Isolation Kit (Stemcell, catalog 19853) according to the manufacturer’s instructions. Next, the CD8^+^ T cells were incubated with IL-2 (10 ng/mL), an anti-CD3 antibody (5 μg/mL), and an anti-CD28 antibody (2.5 μg/mL) for 72 hours. In a different setting, OT-I cells were cultured with OVA-loaded dead tumor cells and IL-2 for 72 hours.

### Induction of BMDMs and bone marrow–derived DCs.

Bone marrow cells were isolated from the hind limbs of the mice. RRCs in the cell suspension were lysed with RBC lysis buffer. BMDMs were generated from bone marrow cells by culturing with M-CSF (20 ng/mL), and the culture medium was changed every 3 days. The macrophages were collected between days 7 and 9 for further experimentation. Bone marrow–derived DCs (BMDCs) were generated from bone marrow cells by culturing with GM-CSF (20 ng/mL) and IL-4 (20 ng/mL). The DCs were labeled with CD11c and sorted using a flow cytometer between days 8 and 10.

### Induction of PBMC-derived DCs and macrophages.

PBMCs were obtained from the blood of healthy donors using Ficoll-based density gradient separation. CD14^+^ monocytes were then purified using magnetic bead–based separation and subsequently cultured in DMEM supplemented with 10% FBS. DCs were generated from monocytes by culturing with GM-CSF (50 ng/mL) and IL-4 (20 ng/mL), with medium replacement every 3 days. On day 6, LPS (100 ng/mL) was added for 24 hours to induce DC maturation. Macrophages were generated from monocytes by culturing with M-CSF (50 ng/mL), medium was replaced every 3 days, and maturity was reached at day 7.

### Antigen presentation and T cell activation assay.

Macrophages were exposed to dead OVA-loaded tumor cells for 48 hours, followed by staining with antibodies specific to the OVA peptide–MHC-I–binding epitope and CD11b to detect the surface presentation of the OVA peptide SIINFEKL on H-2Kb (MHC-I). The fluorescence intensity of the H-2Kb–OVA complex on macrophages was detected using FACS. For the T cell proliferation and activation assays, OT-I cells were labeled with CellTrace Violet (CTV). A total of 4 × 10^4^ macrophages or DCs pretreated with dead OVA-loaded tumor cells were cocultured with 2 × 10^5^ CTV-labeled OT-I cells. Following a 3-day incubation, the cells were harvested and assessed for CTV dilution and measurement of the cytokine expression levels in OT-I cells by FACS.

### Phagocytosis assay.

The macrophages or DCs were incubated with GFP-labeled dead tumor cells at a 1:5 ratio. The median fluorescence intensity (MFI) of GFP in macrophages was analyzed by FACS after gating for CD11b or CD11c.

The macrophages were incubated with dead tumor cells at a 1:5 ratio for 24 hours, followed by a 20-minute pulse with pHrodo-SE–labeled 3 μm latex beads. The cells were then thoroughly washed with cold PBS and incubated for 40 minutes to allow for chase. The MFI of pHrodo Red fluorescence in macrophages was analyzed by FACS after gating on CD11b.

In a similar experimental setup, macrophages were incubated with dead tumor cells at a 1:5 ratio for 24 hours and then with pHrodo-SE beads for 20 minutes, followed by a 30-minute incubation with LysoTracker dye. After extensive washes, the macrophages were fixed with 4% paraformaldehyde, and images of immunofluorescence staining were captured using a confocal microscope. The fluorescence intensity of the LysoTracker dye was quantified using ImageJ software (NIH).

### ELISPOT assay.

Tumor-infiltrating lymphocytes were isolated from tumor tissues of Hepa1-6-OVA tumor–bearing mice. A total of 2.5 × 10^5^ cells were cultured with OVA peptide for 48 hours, followed by incubation with a detection antibody to the Mouse IFN-γ ELISPOT Kit (Dakewe, catalog 2210002) manufacturer’s protocol. The images and spot counts were obtained using an AID ELISPOT Reader (Autoimmun Diagnostika).

### Stable cell line generation.

Plasmids encoding TESC-, CALR-, or TAP1-silencing constructs, TESC or OVA overexpression constructs, or the *Tesc*-D128A point mutation plasmid, along with 2 lentivirus-packaging plasmids (psPAX2 and pMD2.G), were cotransfected into HEK293T cells using Lipofectamine 3000 (Invitrogen, Thermo Fisher Scientific, catalog L3000015). After 24 hours, the supernatant containing the lentivirus was collected. Tumor cells were then infected with lentivirus using polybrene and selected with puromycin. The expression of TESC, CALR, TAP1, and TESC (D128A) was further verified by immunoblotting.

### Culture system for hepatoma cells.

Hepa1-6 or H22 hepatoma cells were treated with BAPTA (10 μM) for 1 hour to chelate cytosolic Ca^2+^. Hepa1-6 or H22 hepatoma cells were treated with TUDCA (100 μM) for 24 hours to inhibit ER stress. Hepa1-6 or H22 hepatoma cells were treated with a continuous concentration gradient of OICR-9429 (0 μM, 70 μM, 120 μM, 140 μM, or 240 μM) for 24 hours to inhibit H3K4me3 or TESC expression in vitro. Hepa1-6 or H22 hepatoma cells were treated with a continuous concentration gradient of isoginkgetin (0 μM, 5 μM, 10 μM, 20 μM, or 40 μM) for 24 hours to inhibit H3K4me1 expression in vitro.

### Data-independent acquisition mass spectrometry analysis.

Whole-cell lysates were prepared from NC or sh*Tesc* H22 cells. The data-independent acquisition mass spectrometry (DIA-MS) analysis was conducted by New Creation Biotechnology. Differentially expressed proteins were identified and subjected to a Gene Ontology (GO) biological process analysis. The criteria for selecting differentially expressed proteins were as follows: a *P* value of less than 0.05 and a log_2_ fold change (FC) of greater than 1.2 or a log_2_ (FC) of less than 0.83. For the analysis of “eat-me” and “don’t-eat-me” signals, the proteins shown in Supplemental Figure 4L were extracted from the proteomics dataset, and the expression levels between 2 groups were visualized as a heatmap.

### RNA-seq and scRNA-seq analyses.

For RNA-seq analysis, total RNA was extracted from tumor biopsy specimens collected from patients with HCC in cohort 3 prior to OXA-based chemotherapy plus ICB treatment and from BMDMs. RNA-seq was conducted by Novogene.

For the characterization of gene expression profiles of BMDMs after engulfment of NC or *Tesc* Hepa1-6 cells, genes showing distinct expression patterns across different gene sets were identified according to fragments per kilobase of transcript per million mapped reads (FPKM) values and visualized using a heatmap.

For the analysis of the CD8^+^ T cell infiltration score, RNA-seq data from TCGA dataset were utilized to assess the infiltration score with the xCell algorithm (54). The samples were divided into low and high infiltration groups on the basis of these scores, and differential gene expression analysis was performed between the 2 groups.

For the identification of the primary cell populations expressing TESC within the TME, the scRNA-seq data (GSE166635) from patients with HCC were analyzed on the TISCH2 database.

For the single-cell sequencing analysis, the GEO dataset GSE151530 was used to assess the correlation between tumor TESC expression and cytotoxic T lymphocyte (CTL) infiltration. Samples were included if the patients were pathologically diagnosed with HCC, had not received prior treatment, and the tissues contained more than 15 tumor cells. Data normalization was performed using the NormalizeData, FindVariableFeatures, and ScaleData functions in the Seurat package. Clustering was performed using FindNeighbors and FindClusters with a resolution of 0.1, followed by the identification of marker genes with FindAllMarkers. On the basis of these marker genes, T cells were subdivided into 6 clusters, which were then used to compare the proportions of CTLs. The Kyoto Encyclopedia of Genes and Genomes (KEGG) enrichment analysis of the differentially expressed genes (DEGs) from CTLs in the high-TESC group was performed using the clusterProfiler package. Analyses of the antigen presentation and phagocytosis scores of macrophages were performed according to the antigen presentation–associated gene set and the phagocytosis-associated gene set (55). The gene set is provided in Supplemental Table 2.

Differential gene expression analysis was performed using the DESeq2 R package. Genes with a *P* value of less than 0.01 and a log_2_ (FC) of greater than1.5 were considered DEGs.

For investigation of the correlation between TESC and the therapeutic efficacy of immunotherapy in patients with HCC, Mendeley Data (skrx2fz79n) were used to stratify responders and nonresponders, and the transcriptional levels of TESC in malignant cells were subsequently analyzed.

### Reagents and primers.

The antibodies used for FACS, IHC, immunofluorescence, immunoblotting, ChIP assays, and animal studies are detailed in Supplemental Tables 3 and 4. The recombinant proteins, peptides, chemicals, and commercial assays utilized in this study are listed in Supplemental Table 5.

### Additional resources.

Figure 1, H–J, M, and O, Supplemental Figure 2, A and C, Supplemental Figure 3, A, B, and D, and Supplemental Figure 6, E, J, and P, were created with BioRender.com. Data graphics were generated using GraphPad Prism 8.0 (GraphPad Software) and R version 4.1.2.

### Statistics.

Data are presented as the mean ± SEM. For comparisons between two groups, 2-tailed Student’s *t* tests were used, while 1-way ANOVA was applied to compare outcomes across multiple groups. For GO and KEGG enrichment analyses, Fisher’s exact test was used to assess significance, and Benjamini-Hochberg correction (BH) was applied for multiple testing. Pearson’s correlation coefficients were calculated to assess correlations between parameters. The Kaplan-Meier method was used to estimate cumulative survival time, and the log rank test was conducted to compare the groups. ROC curve analysis was conducted to predict the response to ICB. Statistical analyses were conducted using GraphPad 8.0 Prism, with a *P* value of less than 0.05 considered statistically significant.

### Study approval.

All the mice were housed under specific pathogen–free conditions in the animal facility of Sun Yat-sen University Cancer Center. All animal experiments were performed in accordance with the NIH *Guide for the Care and Use of Laboratory Animals* (8th edition, National Academies Press) and were approved by the Sun Yat-sen University Cancer Center Ethics Committee (ethics approval no. L025503202202009). Written informed consent was obtained from each patient, and the study protocol was approved by the Ethics Review Committee of Sun Yat-sen University Cancer Center (ethics approval no. G2025-107).

### Data availability.

All supporting data values are available in the Supporting Data Values file. The raw sequencing data reported in this article have been submitted to the Genome Sequence Archive (https://ngdc.cncb.ac.cn/gsa/browse/CRA037701) and the Genome Sequence Archive for Human (https://ngdc.cncb.ac.cn/gsa-human/browse/HRA016607). The raw data for DIA-MS have been submitted to the Open Archive for Miscellaneous Data (OMIX) (https://ngdc.cncb.ac.cn/omix/release/OMIX014731).

## Author contributions

All authors have full access to all data used in the study and take responsibility for the integrity of the data and the accuracy of the data analysis. ZGZ, DPC, and MZ conceptualized the study and supervised the project, determining the order of corresponding authors. JLW, JCW, and YXP developed the methodology and performed the validation. MRH and ZKZ conducted the formal analysis. HZ, TQW, YHZ, ZLH, YZF, and WP carried out the experiments. LX, YJZ, MSC, and DDH provided the resources. ZYY and JBC curated the data. ZGZ, DPC, JLW, and JCW wrote the manuscript. MRH and JLW prepared the visualizations. ZGZ, DPC, and JCW acquired the funding. The order of the co–first authors was determined on the basis of their relative efforts and contributions to the study.

## Conflict of interest

The authors have declared that no conflict of interest exists.

## Funding support

- National Natural Science Foundation of China grant 82473444 (to ZGZ).
- National Natural Science Foundation of China grant 32370970 (to DPC).
- National Natural Science Foundation of China grant 82303893 (to JCW).
- Natural Science Foundation of Guangdong Province grant 2025B1515020016 (to DPC).
- Natural Science Foundation of Guangdong Province grant 2025A1515012405 (to ZGZ).
- Natural Science Foundation of Guangdong Province grant 2024A1515012966 (to ZGZ).
- Beijing Kechuang Medical Development Foundation grant KC2023-JX-0186-FZ101 (to ZGZ).
- Beijing Weiai Public Welfare Foundation grant YCKY-20240150326010 (to ZGZ).

## Supplementary Material

Supplemental data

Unedited blot and gel images

Supporting data values

## Version 1 04/02/2026

In-Press Preview

## Version 2 06/01/2026

Electronic publication

## Version 3 10/02/2026

Added co-first author statement
