# Supplementary material for: Tescalcin is a phagocytic checkpoint driving immune escape and limiting immunotherapeutic efficacy in hepatocellular carcinoma
Source: J Clin Invest. 2026 Apr 2;136(11):e200415. doi: 10.1172/JCI200415 (PMC13221238; doi:10.1172/JCI200415)
Supplement: Supplemental data [file jci-136-200415-s133.pdf]

Supplementary Material

Supplement to: Tescalcin is a phagocytic checkpoint driving immune escape and limiting immunotherapeutic efficacy in hepatocellular carcinoma

Content

Supplemental tables.. 2

Supplemental figures and figure legends 7

Supplemental tables

Supplemental Table 1. Clinical characteristics of 465 hepatocellular carcinoma patients

Patient characteristics	Cohort 1	Cohort 2 TCGA database	Cohort 3	Cohort 4
Number	14	401	30	20
Age, y, median (range)	48 (35–68)	61 (16–90)	50 (30–68)	49 (30–62)
Sex, male / female, n	14 / 0	271 / 130	26 / 4	18 / 2
HbsAg, positive / negative, n	14 / 0	154 / 247	29 / 1	20 / 0
ALT, U/L, median (range)	45.3 (25.8–162.8)	/	41.8 (32.2–162.8)	44.0 (30.4–160.4)
AFP (≤ 25 ng/mL / > 25 ng/mL), n	3 / 11	177 / 129	6 / 24	4 / 16
Tumor multiplicity, solitary / multiple, n	9 / 5	193 / 205	19 / 11	13 / 7
Tumor size (≤ 5 cm / > 5 cm), n	0 / 14	/	0 / 30	0 / 20
Vascular invasion, absent / present, n	9 / 5	113 / 227	25 / 5	17 / 3
TNM stage, I + II / III + IV, n	7 / 7	178 / 82	19 / 11	13 / 7

Abbreviations: AFP, alpha-fetoprotein; ALT, alanine aminotransferase; HbsAg, hepatitis B surface antigen; TNM, Tumor Node Metastasis.

Supplemental Table 2. Antigen presentation and phagocytosis-associated gene set

Antigen presentation-associated genes						
B2M	TAP1	TAPBPL	CALR	PSMB9	PSMB10	ERAP1
PDIA3	NLRC5	RFX5	PSME1	PSME2	PSME3	CIITA
HSP90AB1	HSP90AA1	HSP90B1				
Phagocytosis-associated genes						
CD36	CD14	MFGE8	MRC2	MARCO	PDCD5	RAB7A
LAMP1	ATP6V0C	FCGR1B	MERTK	AXL	TIMD4	ELMO1
RAC1	SYK	FCGR1A				

Supplemental Table 3. Antibodies for flow cytometry

Antibodies	Source	Clone	Identifier
Anti-Mouse-F4/80-Brilliant Violet 421	BD Biosciences	T45-2342	565411
Anti-Mouse-CD45-Alexa Fluor® 700	BioLegend	30-F11	103128
Anti-Mouse-CD3ε-FITC	BioLegend	145-2C11	100306
Anti-Mouse-CD8α-APC	BioLegend	HIT8a	300912
Anti-Mouse-Granzyme B-Brilliant Violet 421	BioLegend	QA18A28	396413
Anti-Mouse-IFN-γ-PE	BioLegend	XMG1.2	505807
Anti-Mouse-TNF-α-PE/Cyanine7	BioLegend	MP6-XT22	506323
Anti-Mouse-Ki-67-PE	BioLegend	11F6	151210
Anti-Mouse-CD11c-PE	BioLegend	N418	117307
Anti-Mouse-H-2Kb bound to SIINFEKL-PE/Cyanine7	BioLegend	25-D1.16	141607
Anti-Mouse-H-2Kb-Alexa Fluor® 647	BioLegend	28-8-6	114612
Anti-Mouse-CD11b-FITC	BioLegend	M1/70	101206
Anti-Mouse-CD47-FITC	BioLegend	miap301	127503
Anti-Human-HLA-ABC-PE	BioLegend	W6/32	311405
Anti-Human-HLA-DR-APC	BioLegend	L243	307609
Anti-Human-CD11c-Alexa Fluor® 700	BioLegend	Bu15	337219
Anti-Human-CD14-Alexa Fluor® 700	BioLegend	63D3	367113
Anti-Human-CD45-PerCP-Cy5.5	BD Biosciences	HI30	564105
Anti-Human-CD3-APC	BD Biosciences	UCHT1	555335
Anti-Human-CD8- Brilliant Violet 510	BD Biosciences	RPA-T8	563256
Anti-Human-IFN-γ-FITC	BD Biosciences	B27	554700
Anti-Human- Granzyme B- PE	BD Biosciences	GB11	561142
Zombie NIR	BioLegend	N/A	423106

Supplemental Table 4. Antibodies for immunohistochemistry, immunofluorescence, and immunoblotting, and animal study

Antibodies	Source	Clone	Identifier
Anti-Human/Mouse-TESC	Proteintech	N/A	11125-1-AP
Anti-Human/Mouse-α-Tubulin	Proteintech	1E4C11	66031
Anti-Human/Mouse-p-IRE1α	ABclonal	N/A	AP0878
Anti-Human/Mouse-p-PERK	ABclonal	N/A	AP0886
Anti-Human/Mouse-CALR	abcam	EPR3924	ab92516
Anti-Human/Mouse-H3K4me1	abcam	ERP16597	Ab176877
Anti-Human/Mouse-H3K4me3	abcam	EPR20551-225	ab213224
Anti-Human/Mouse-Granzyme B	abcam	EPR22645-206	ab255598
Anti-Human-CD68	abcam	KP1	ab955
Anti-Human-CD45	STARTER	SDT-R035	S0B2087
Anti-Human-CD8α	MCE	N/A	HY-P80607
Anti- Human-pan-CK	Cell Signaling Technology	C11	4545
Anti-Human-MHC-I	Cell Signaling Technology	EMR8-5	88274
Anti-Mouse-MHC-I	Cell Signaling Technology	E8E7N	76828
Anti-Mouse-CD8α	Cell Signaling Technology	D4W2Z	98941
Anti-Mouse-CD4	Cell Signaling Technology	D7D2Z	25229
Anti-Mouse-Ly-6G	Cell Signaling Technology	E6Z1T	87048
Anti-Mouse-F4/80	Cell Signaling Technology	D2S9R	70076
Anti-Mouse-BIP	Cell Signaling Technology	C50B12	3177
Anti-Mouse-p-eIF2α	Cell Signaling Technology	D9G8	3398
Anti-Mouse-eIF2α	Cell Signaling Technology	D7D3	5324
Anti-Mouse-NKp46	abcam	EPR23097-35	ab233558
Anti-Mouse-MHC-II	abcam	MRC OX-6	ab23990
Anti-Mouse-IRE1α	ABclonal	N/A	A17940
Anti-Mouse-XBP1	ABclonal	N/A	A25319
Anti-Mouse-PERK	ABclonal	N/A	A18196
Anti-Mouse-CHOP	ABclonal	N/A	A20987
Anti-Mouse-TAP1	Proteintech	N/A	11114-1-AP
Anti-Mouse-CD47	Proteintech	1E1D8	66304-1
Anti-Mouse-Vinculin	Proteintech	2B5A7	66305-1
Anti-Mouse-B220	BD Biosciences	RA3-6B2	557390
Anti-GFP	MCE	YA415	HY-P80141
Fluorescent secondary antibody (Alexa Fluro 488)	ThermoFisher	N/A	A11034
Anti-mouse CSF1R	Bio X Cell Life Sciences	AFS98	BE0213
Rat IgG2a Isotype	Bio X Cell Life Sciences	2A3	BE0089
Anti-mouse CD8α	Bio X Cell Life Sciences	2.43	BE0061
Rat IgG2b Isotype	Bio X Cell Life Sciences	LTF-2	BE0090
Anti-mouse PD-1	STARTER	S-5001	S0B0594
Invivo mouse IgG1 isotype	STARTER	S-844-79HL	S0B0760
Ultra-LEAF™ Purified anti-mouse CD3ε	BioLegend	145-2C11	100340
Ultra-LEAF™ Purified anti-mouse CD28	BioLegend	37.51	102116

Supplemental Table 5. Recombinant proteins, chemicals, and critical commercial assays

Name	Source	Identifier
Recombinant proteins		
M-CSF (human)	Sino Biological	11792-HNAH
M-CSF (mouse)	PeproTech	315-02
GM-CSF (human)	NovoProtein	C003
GM-CSF (mouse)	PeproTech	315-03
IL-4 (human)	NovoProtein	CX03
IL-4 (mouse)	PeproTech	214-14
IL-2	PeproTech	212-12
Ovalbumin peptide (257-264)	MCE	HY-P1489
Chemicals		
TUDCA	MCE	HY-19696
BAPTA	MCE	HY-100545
OICR-9429	MCE	HY-16993
H2DCFDA	MCE	HY-D0940
Isoginkgetin	MCE	HY-N2117
Oxalipaltin	MCE	HY-17371
LPS	MCE	HY-D1056
Mag-Fluo-4-AM	AAT Bioquest	20401
Rhod2-AM	AAT Bioquest	21060
Critical commercial assays		
Mouse IFN-γ ELISA kit	MultiSciences	70-EK280/3
Mouse IFN-γ ELISPOT kit	Dakewe	2210002
Apoptosis detection kit	GOONIE	100-102
Mouse tumor dissociation kit	RWD	DHTE-5001
Fixation/permeabilization kit	BD Biosciences	554714
Transcription factor buffer kit	BD Biosciences	562574
Pierce ECL kit	NCM Biotech	P10300
Anti-CD14 magnetic beads	Miltenyi	130-050-201
PANO 7-plex IHC kit	Panovue	10004100050
EasySep mouse $CD8^+$ T Cell isolation kit	Stemcell	19853
EZ-Magna ChIP kit	Sigma-Aldrich	17-10086
pHrodo™ Red, SE	Invitrogen	P36600
Lysotracker dye	Invitrogen	L12492
Trizol Reagent	Invitrogen	AM9738
Cell stimulation cocktail	ThermoFisher	00-4975-03
CellTrace™ Violet	ThermoFisher	C34554
Lipofectamine™ 3000	ThermoFisher	L3000015
Matrigel	MCE	HY-K6005
Cell Counting Kit-8	MCE	HY-K0301
Puromycin	MCE	HY-B1743
Polybrene	MCE	HY-112735
RIPA Lysis buffer	NCM Biotech	WB3100
SYBR Green Real-Time PCR Master Mix	Solarbio	A8010
Red blood cell lysis buffer	absin	abs9241
Other		
DMEM	Gibco	C11885500BT
RPMI 1640	Gibco	C11875500BT
Fetal bovine serum	Bio-Channel	BC-SE-FBS07C
Penicillin-streptomycin	NCM Biotech	C100C5
Cell culture dish	SAINING Life Science	1022000
Cell staining buffer	BioLegend	420201
DAPI	MCE	HY-K1047
BSA	Biofroxx	4240GR250
Tissue fixative	LEAGENE	DF0111

Supplemental figures and figure legends

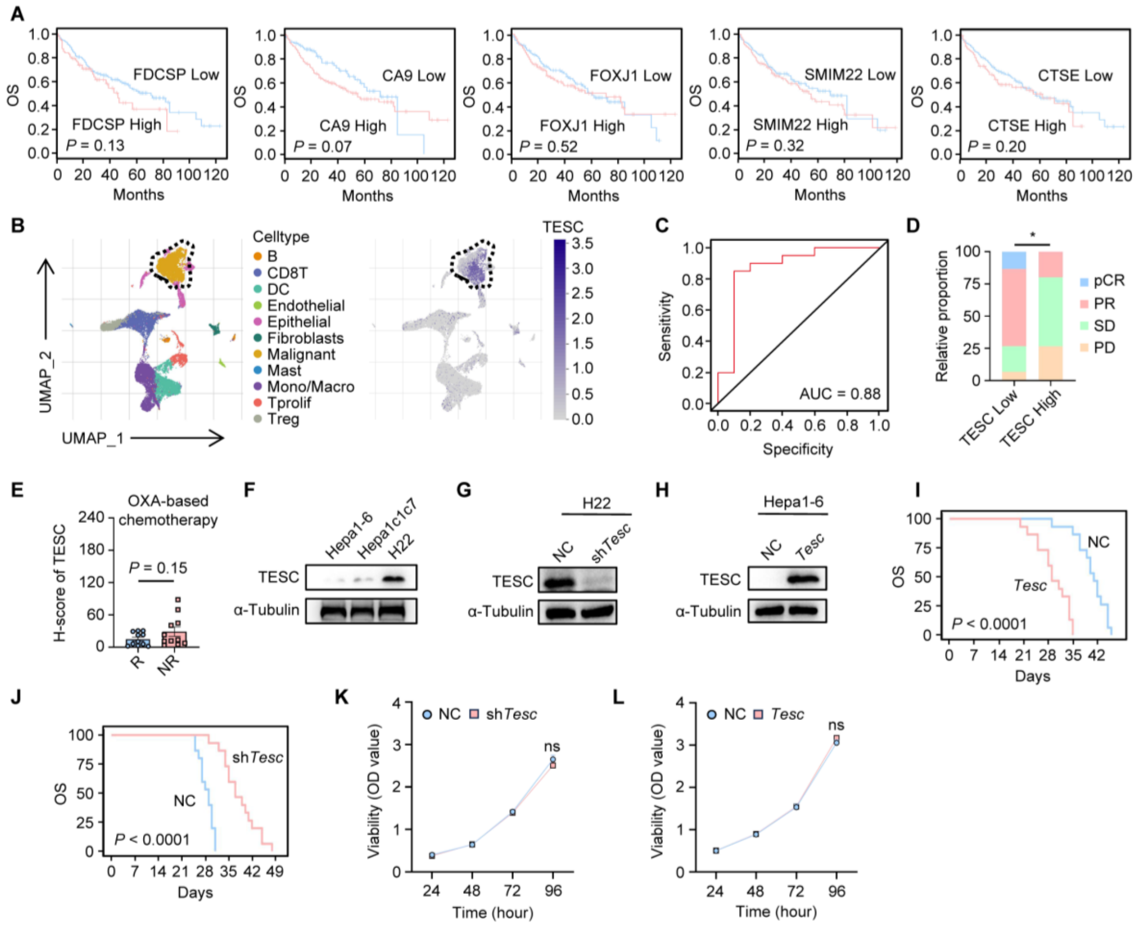

Supplemental Figure 1. Tumor-intrinsic TESC contributes to resistance to antitumor immunity and immunotherapy, related to Figure 1. (A) Correlations between transcript levels of FDCSP, CA9, FOXJ1, CTSE, and SMIM22 and the OS of HCC patients from TCGA dataset. Patients were grouped by the median expression of the indicated genes. Log-rank test. (B) Analysis of TESC expression in HCC using the single-cell RNA sequencing data set (GSE166635) from the TISCH database (http://tisch.comp-genomics.org/). (C) ROC curve showing the predictive performance of intratumoral TESC expression for response to oxaliplatin-based chemotherapy plus sintilimab therapy. The area under the curve (AUC) was 0.88. (D) Stacked bar plot showing the clinical efficacy of combined therapy in patients from cohort 3 (n = 30). 2-tailed Student's t test. (E) The levels of TESC in HCC tissues from oxaliplatin-based chemotherapy responders and nonresponders (cohort 4) (n = 20). 2-tailed Student's t test. (F) The intrinsic expression levels of TESC in several murine HCC cell lines were analyzed by immunoblotting. (G and H) The knock down efficiency of TESC in TESC-silenced (sh*Tesc*) H22 cells and the overexpression efficiency of TESC in TESC-overexpressing (*Tesc*) Hepa1-6 cells were analyzed by immunoblotting. (I and J) The survival time of mice bearing sh*Tesc* H22 hepatoma or *Tesc* Hepa1-6 hepatoma was assessed (n = 15). Log-rank test. (K and L) Effect of *Tesc* on proliferation of Hepa1-6 cells and sh*Tesc* on proliferation of H22 cells (n = 3). 2-tailed Student's t test. ns, not significant, *P < 0.05. HCC, hepatocellular carcinoma; NR, nonresponder; OS, overall survival; pCR, pathological complete response; PD, progressive disease; PR, partial response; R, responder; ROC, receiver operating characteristic; SD, stable disease.

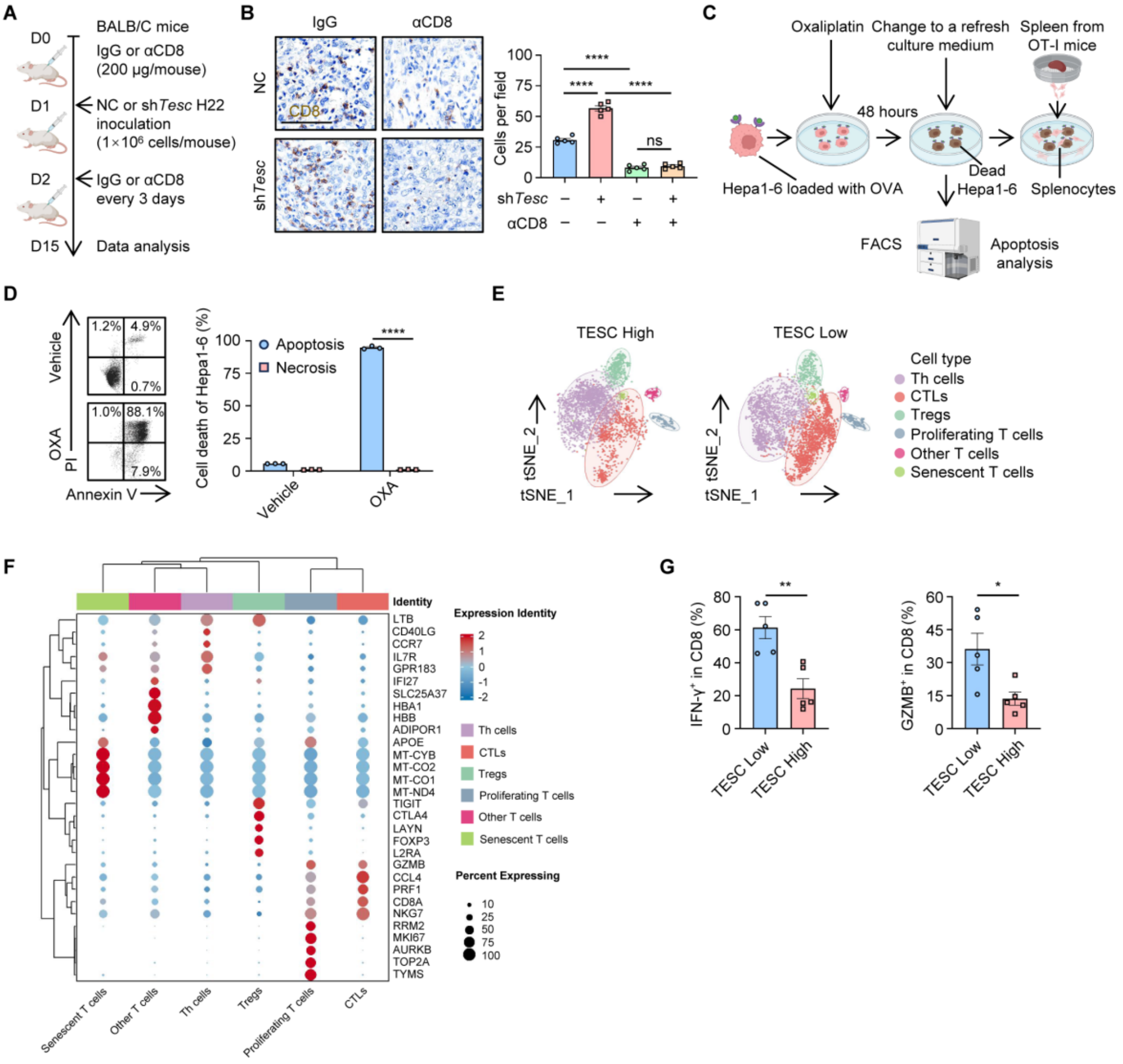

Supplemental Figure 2. Tumor TESC impairs CD8^{+} T-cell–mediated antitumor immunity, related to Figure 2. (A) NC or sh*Tesc* hepatoma-bearing mice were untreated or injected with isotype or αCD8 antibody as described. (B) The effect of αCD8 antibody on the CD8^{+} T cell infiltration in mouse tumor tissues were analyzed (n = 5). Scale bar, 80 μm. 1-way ANOVA analysis. (C) The apoptosis levels of oxaliplatin-induced cell death in Hepa1-6 cells were analyzed by FACS. OT-I splenocytes were cultured with oxaliplatin-induced dead OVA-loaded NC or *Tesc* Hepa1-6 cells for 3 days. (D) FACS analysis of apoptosis levels in Hepa1-6 cells treated with oxaliplatin for 48 hours (n = 3). 2-tailed Student's t test. (E) t-SNE plot showing T-cell clusters (GSE151530). Patients were grouped by the median tumor TESC expression in tumor cells. (F) Dot plot showing expression of selected marker genes across different cell types from the single-cell RNA sequencing (GSE151530). The size of the dot represents the proportion of cells that expressed the genes, and its color encodes the average expression level. (G) The CTL functions within tumor tissues of HCC patients were analyzed. Patients were grouped by the median TESC expression, as determined by immunohistochemistry. 2-tailed Student's t test. ns, not significant, *P < 0.05, ** P < 0.01, and **** P < 0.0001. CTL, cytotoxic T lymphocyte; FACS, flow cytometry; OVA, ovalbumin; OXA, oxaliplatin.

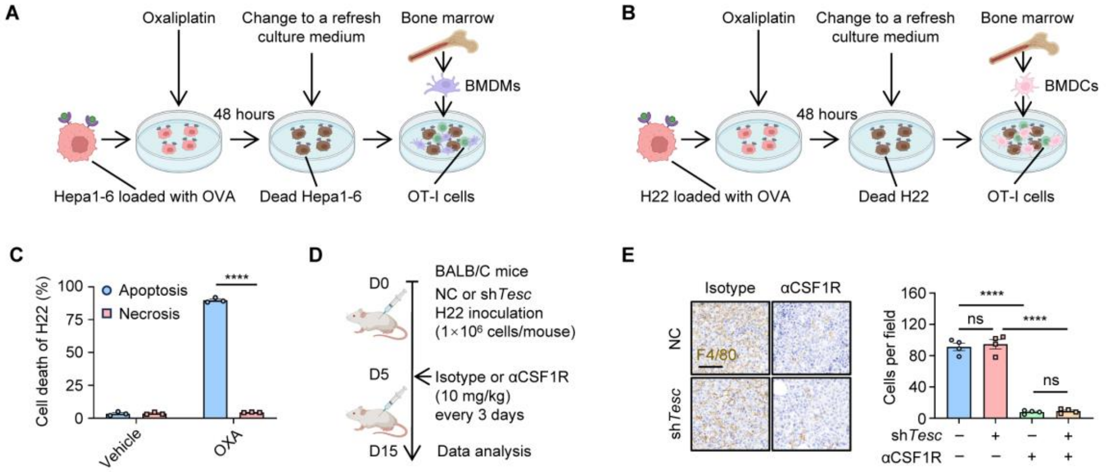

Supplemental Figure 3. TESC attenuates tumor immunogenicity by disrupting antigen presentation by APCs, related to Figure 3. (A) OT-I cells were cultured with oxaliplatin-induced dead OVA-loaded NC or *Tesc* Hepa1-6 cells in the presence or absence of BMDMs for 3 days. (B) OT-I cells were cultured with oxaliplatin-induced dead OVA-loaded NC or sh*Tesc* H22 cells in the presence or absence of BMDCs for 3 days. (C) FACS analysis of apoptosis levels in H22 cells treated with oxaliplatin for 48 hours (n = 3). 2-tailed Student's t test. (D) NC or sh*Tesc* hepatoma-bearing mice were untreated or injected with isotype or αCSF1R antibody as described. (E) The effect of αCSF1R antibody on the macrophage infiltration in mouse tumor tissues were analyzed (n = 4). Scale bar, 50 μm. 1-way ANOVA analysis. ns, not significant, and **** $P < 0.0001$. APC, antigen presentation cell; BMDC, bone marrow–derived dendritic cell; BMDM, bone marrow–derived macrophage.

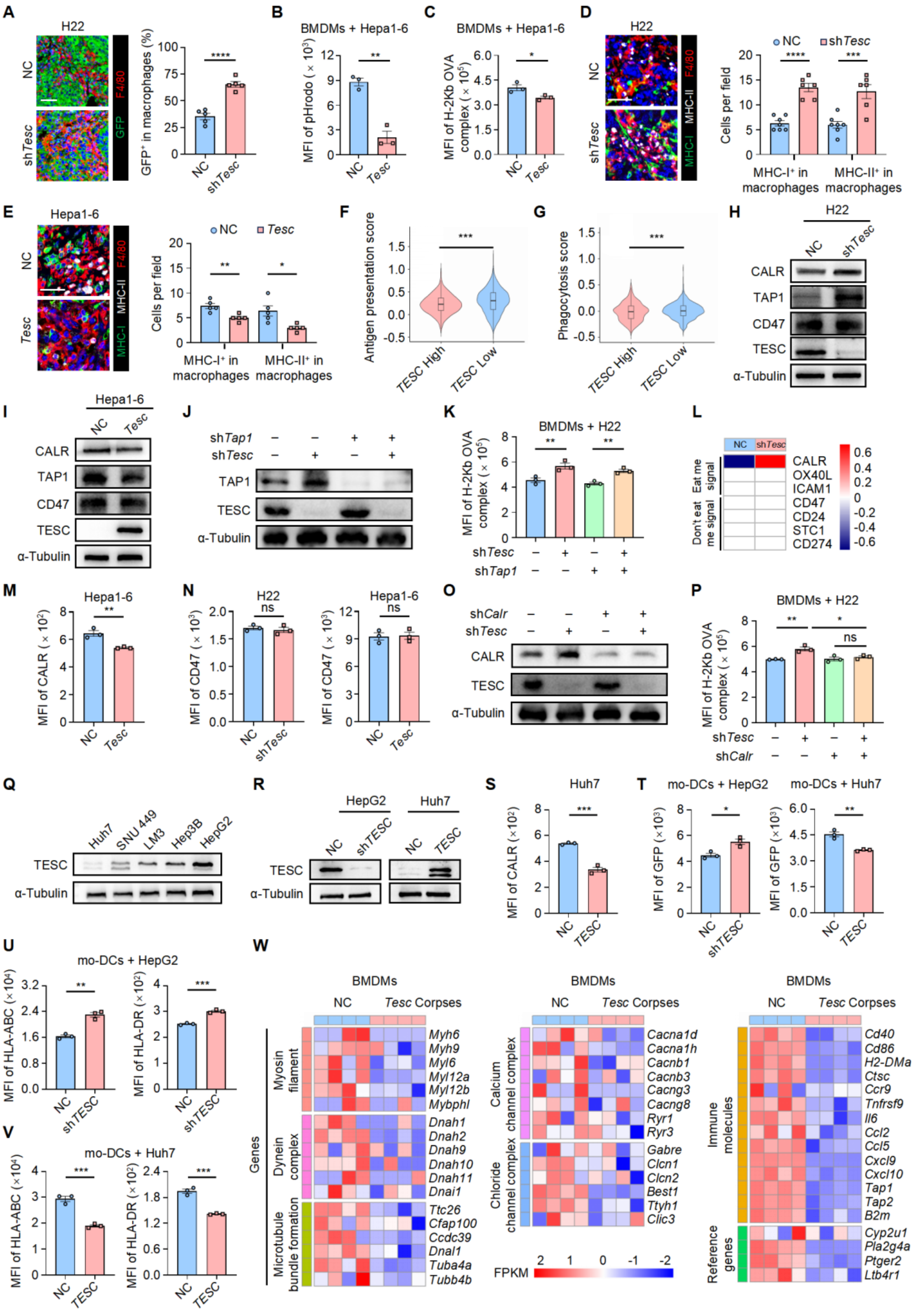

Supplemental Figure 4. TESC restrains CALR membrane translocation to inhibit macrophage function, related to Figure 4. (A) Immunofluorescence analysis of GFP^+ macrophage infiltration in sh*Tesc* H22 hepatomas (*n* = 5). Scale bar, 50 µm. 2-tailed Student's t test. (B) BMDMs were cultured with dead *Tesc* Hepa1-6 cells for 20 hours, followed by pHrodo Red FACS analysis (*n* = 3). 2-tailed Student's t test. (C) FACS analysis of H-2Kb–OVA complexs in BMDMs cultured with dead OVA-loaded *Tesc* Hepa1-6 cells for 48 hours (*n* = 3). 2-tailed Student's t test. (D and E) Immunofluorescence analysis of $MHC\text{-}I^+$ and $MHC\text{-}II^+$ macrophage infiltration in sh*Tesc* H22 or *Tesc* Hepa1-6 hepatoma (*n* = 5–7). Scale bar, 50 µm. 2-tailed Student's t test. (F and G) Antigen presentation and phagocytosis score in macrophages from HCC patients (GSE151530) grouped by median TESC expression. 2-tailed Student's t test. (H and I) Immunoblot analysis of CALR, TAP1, CD47 and TESC in sh*Tesc* H22 or *Tesc* Hepa1-6 cells. (J) Immunoblot analysis of TAP1 expression in TAP1-silenced (sh*Tap1*)-transfected sh*Tesc* H22 cells. (K) FACS analysis of H-2Kb–OVA in BMDMs cultured with dead OVA-loaded sh*Tap1*-transfected sh*Tesc* H22 cells for 48 hours (*n* = 3). 1-way ANOVA analysis. (L) "Eat me" signal and "don't eat me" signals enriched in sh*Tesc* H22 cells by DIA-MS. (M) FACS analysis of CALR expression in *Tesc* Hepa1-6 cells (*n* = 3). 2-tailed Student's t test. (N) FACS analysis of CD47 expression in sh*Tesc* H22 or *Tesc* Hepa1-6 cells (*n* = 3). 2-tailed Student's t test. (O) Immunoblot analysis of CALR expression in CALR-silenced (sh*Calr*)-transfected sh*Tesc* H22 cells. (P) FACS analysis of H-2Kb–OVA in BMDMs cultured with dead OVA-loaded sh*Calr*-transfected sh*Tesc* H22 cells for 48 hours (*n* = 3). 1-way ANOVA analysis. (Q) Immunoblot analysis of TESC expression in several human HCC cell lines. (R) Immunoblot analysis of TESC expression in sh*TESC* HepG2 cells and *TESC* Huh7 cells. (S) FACS analysis of CALR expression in *TESC* Huh7 cells (*n* = 3). 2-tailed Student's t test. (T–V) FACS analysis of GFP, HLA-ABC, and HLA-DR in DCs cultured with dead GFP-labeled sh*TESC* HepG2 or *TESC* Huh7 cells. (*n* = 3). 2-tailed Student's t test. (W) Heatmap showing representative differentially expressed gene clusters in macrophages engulfing *Tesc* hepa1-6 cells identified by RNA sequencing based on FPKM values. ns, not significant, * $P < 0.05$, ** $P < 0.01$, *** $P < 0.001$, and **** $P < 0.0001$. DC, dendritic cell.

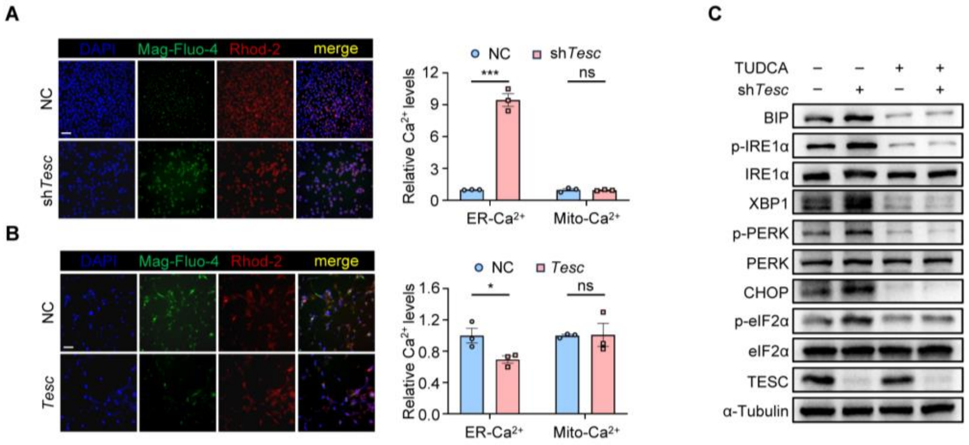

Supplemental Figure 5. TESC regulates CALR surface exposure by modulating calcium homeostasis and attenuating ER stress, related to Figure 5. (A and B) Representative images of immunofluorescence staining for Mag-Fluo-4 and Rhod-2 in NC/sh*Tesc* H22 cells (A) or NC/*Tesc* Hepa1-6 cells (B) (n = 3). Scale bar, 100 μm. The calcium level of ER and mitochondria were analyzed. 2-tailed Student's t test. (C) The activation of ER stress signaling pathways in NC or sh*Tesc* H22 cells treated with TUDCA were analyzed by immunoblotting. ns, not significant, * $P < 0.05$, and *** $P < 0.001$. ER, endoplasmic reticulum; Mito, mitochondria.

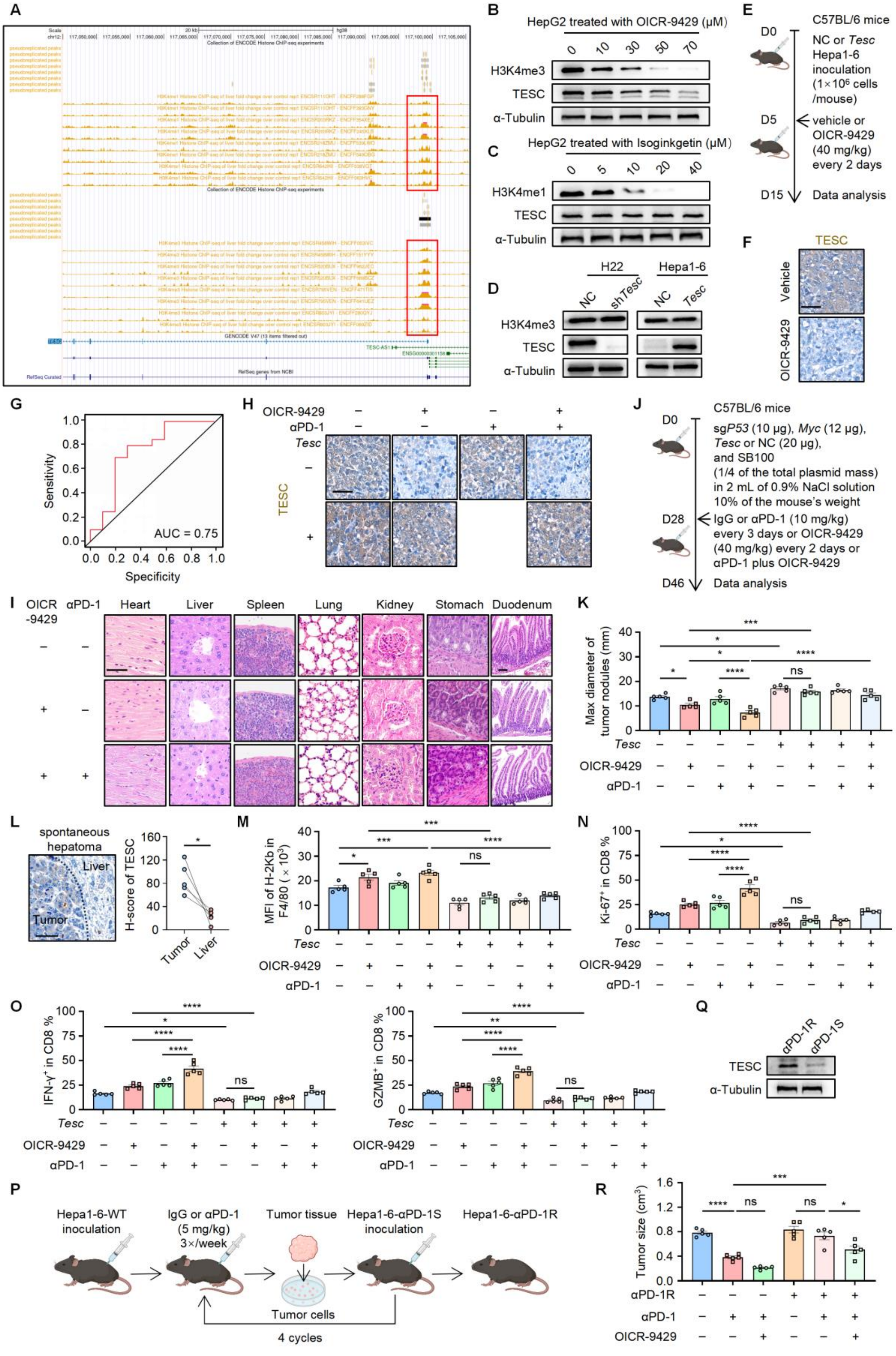

A
B
HepG2 treated with OICR-9429 (μM)
H3K4me3
TESC
α-Tubulin
C
HepG2 treated with Isoginkgetin (μM)
H3K4me1
D
H22
Hepa1-6
E
C57BL/6 mice
NC or Tesc Hepa1-6 inoculation (1×10^6 cells /mouse)
vehicle or OICR-9429 (40 mg/kg) every 2 days
Data analysis
F
Vehicle
OICR-9429
G
Sensitivity
Specificity
AUC = 0.75
H
αPD-1
J
sgP53 (10 μg), Myc (12 μg), Tesc or NC (20 μg), and SB100 (1/4 of the total plasmid mass) in 2 mL of 0.9% NaCl solution 10% of the mouse's weight
IgG or αPD-1 (10 mg/kg) every 3 days or OICR-9429 (40 mg/kg) every 2 days or αPD-1 plus OICR-9429
I
Heart
Liver
Spleen
Lung
Kidney
Stomach
Duodenum
K
Max diameter of tumor nodules (mm)
L
spontaneous hepatoma
Tumor
H-score of TESC
M
MFI of H-2Kb in F4/80 (×10^3)
N
Ki-67+ in CD8 %
O
IFN-γ+ in CD8 %
GZMB+ in CD8 %
Q
αPD-1R
αPD-1S
P
Hepa1-6-WT inoculation
IgG or αPD-1 (5 mg/kg) 3×/week
Tumor tissue
Hepa1-6-αPD-1S inoculation
Hepa1-6-αPD-1R
Tumor cells
4 cycles
R
Tumor size (cm^3)

Supplemental Figure 6. Targeting H3K4me3-induced TESC overcomes immune suppression and potentiates the efficacy of PD-1 combination therapy in HCC, related to Figure 6. (A) ChIP-sequence in human liver tissues revealed binding peaks for H3K4me1 and H3K4me3 on the promoters of TESC from UCSC Genome Browser (ENCSR000FYQ). (B and C) Immunoblot analysis of TESC and H3K4me3 or H3K4me1 expression in HepG2 cells treated with OICR-9429 or Isoginkgetin for 24 hours. (D) Immunoblot analysis of H3k4me3 expression in sh*Tesc* H22 or *Tesc* Hepa1-6 cells. (E) *Tesc* hepatoma-bearing mice were untreated or injected with vehicle or OICR-9429 as described. (F) The effect of OICR-9429 on TESC expression in H22 hepatoma were analyzed by immunochemistry. Scale bar, 50 μm. (G) ROC curve showing the predictive performance of intratumoral H3K4me3 expression for response to oxaliplatin-based chemotherapy plus sintilimab therapy. The area under the curve (AUC) was 0.75. (H) The effect of OICR-9429 on TESC expression in *Tesc* H22 hepatoma were analyzed by immunochemistry. Scale bar, 50 μm. (I) Representative H&E staining images of the indicated organs from mice treated with OICR-9429 and/or anti-PD-1 antibody. Scale bar, 40 μm. (J and K) C57BL/6 mice with spontaneous hepatoma were treated as described above (n = 5). 1-way ANOVA analysis. (L) Representative images of immunohistochemistry for TESC in spontaneous hepatoma tissues and liver tissues ($n = 5$). Scale bar, 50 μm. Paired Student's t test. (M–O) Spontaneous hepatoma-bearing mice were untreated or injected with OICR-9429, the αPD-1 antibody or their combination ($n = 5$). The H-2Kb level in macrophages (M) and CTL function (N and O) were analyzed. 1-way ANOVA analysis. (P) Schematic diagram illustrating the experimental workflow for establishing an anti-PD-1–resistant Hepa1-6 tumor model in mice. (Q) Immunoblot analysis of TESC expression in αPD-1-resistant or -sensitive Hepa1-6 cells. (R) αPD-1-resistant or -sensitive Hepa1-6 hepatoma-bearing mice were untreated or injected with OICR-9429 with or without αPD-1 antibody (n = 5). 1-way ANOVA analysis. ns, not significant, *$P < 0.05$, **$P < 0.01$, ***$P < 0.001$, and ****$P < 0.0001$. PD-1R, anti-PD-1-resistant; PD-1S, anti-PD-1-sensitive; WT, wildtype.
