# Supplementary material for: Tescalcin is a phagocytic checkpoint driving immune escape and limiting immunotherapeutic efficacy in hepatocellular carcinoma
Source: J Clin Invest. 2026 Apr 2;136(11):e200415. doi: 10.1172/JCI200415 (PMC13221238; doi:10.1172/JCI200415)

Full unedited blot for Figure 5A

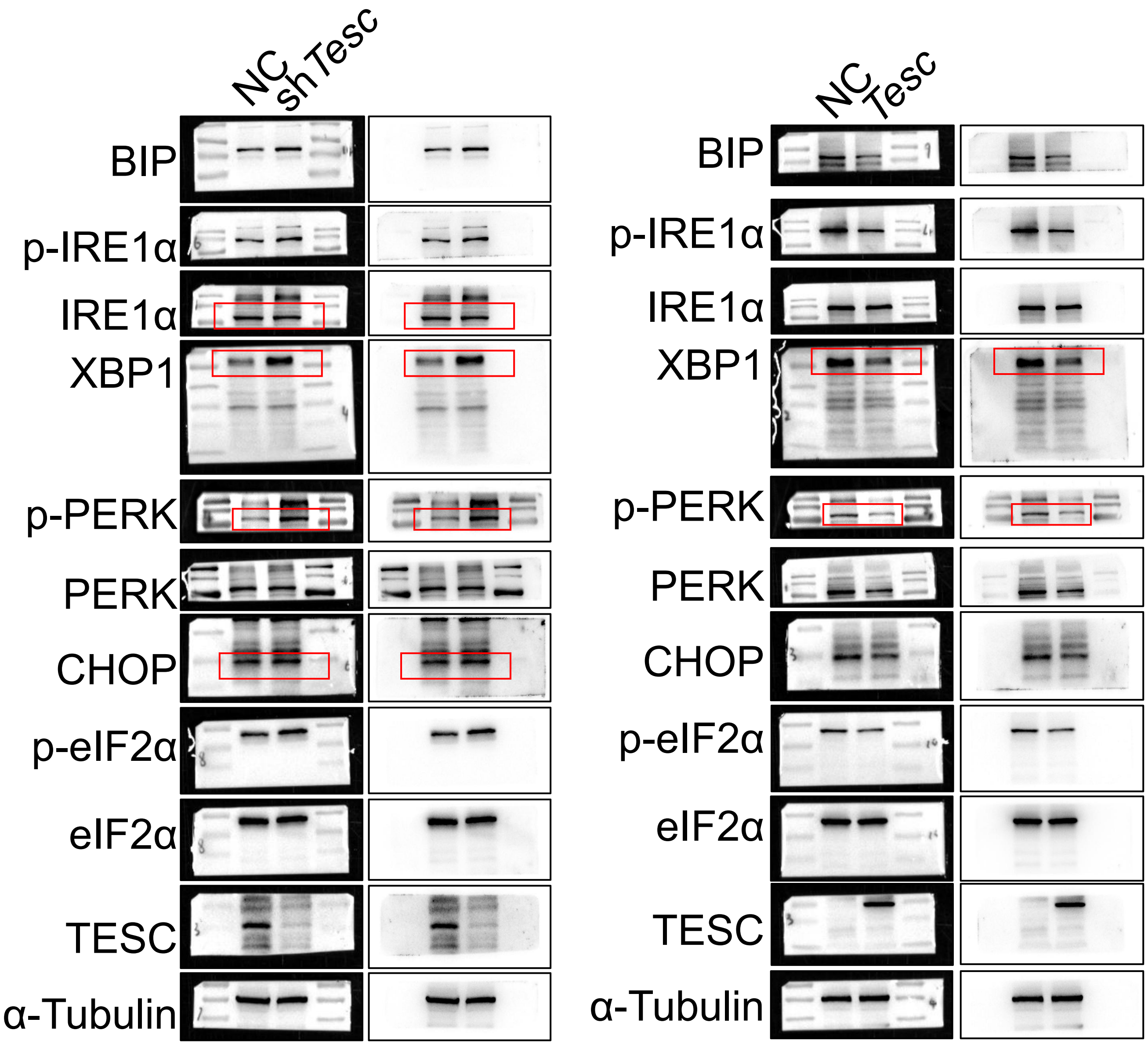

Full unedited blot for Figure 5D

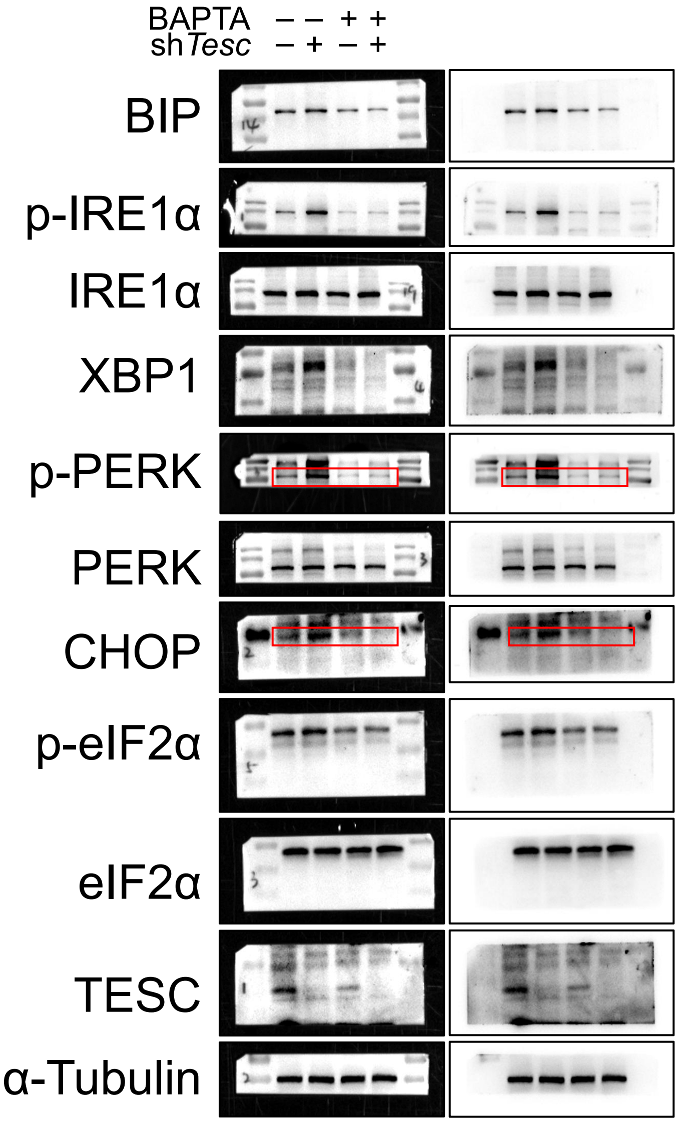

Full unedited blot for Figure 5I

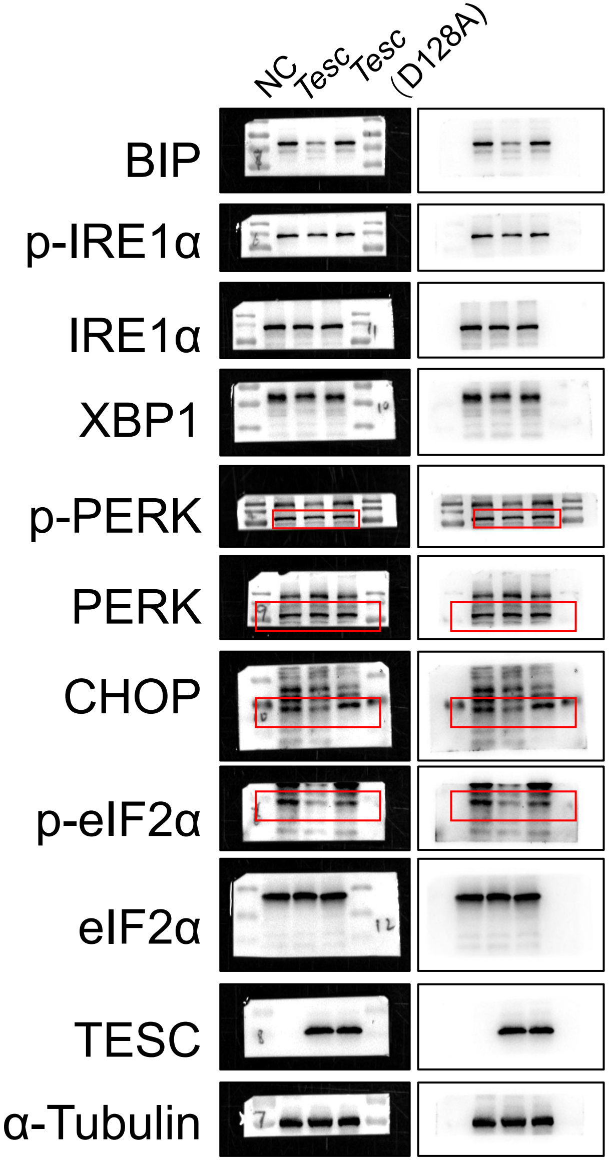

Full unedited blot for Figure 6C

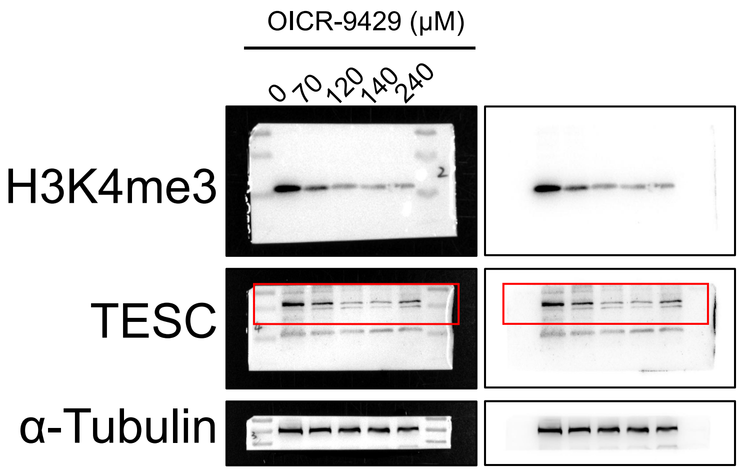

Full unedited blot for Figure 6D

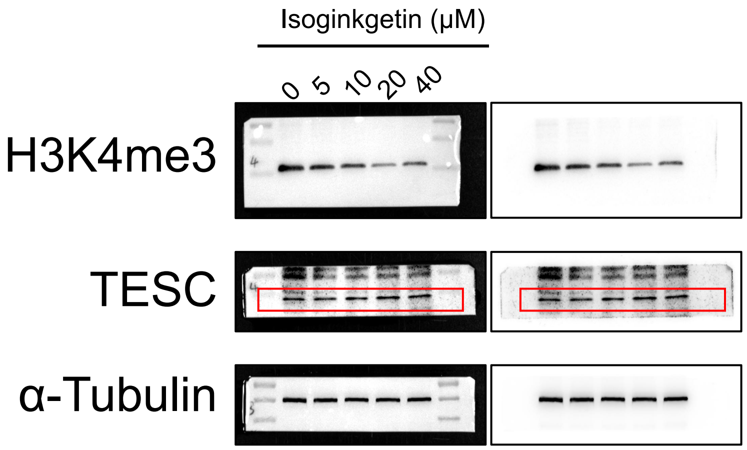

Full unedited blot for Figure S1F

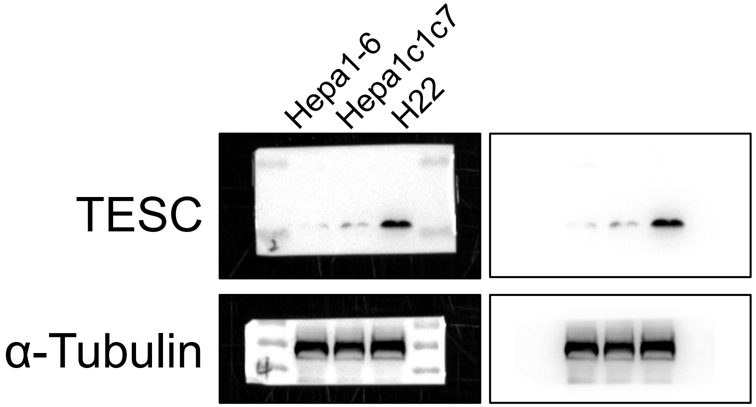

Full unedited blot for Figure S1G

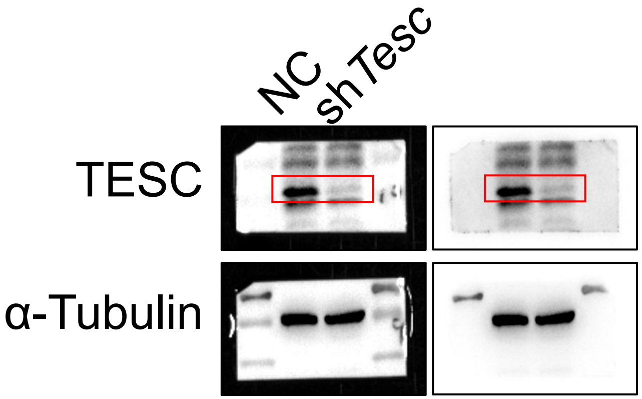

Full unedited blot for Figure S1H

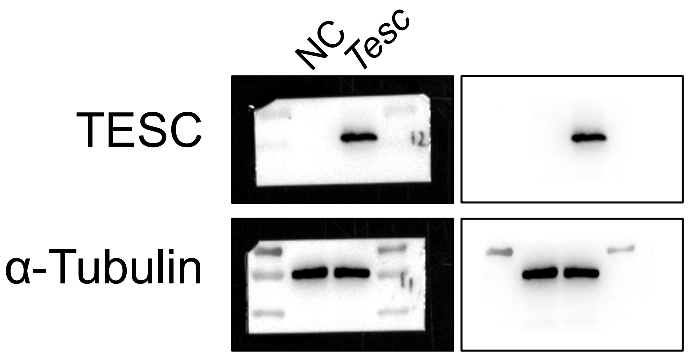

Full unedited blot for Figure S4H

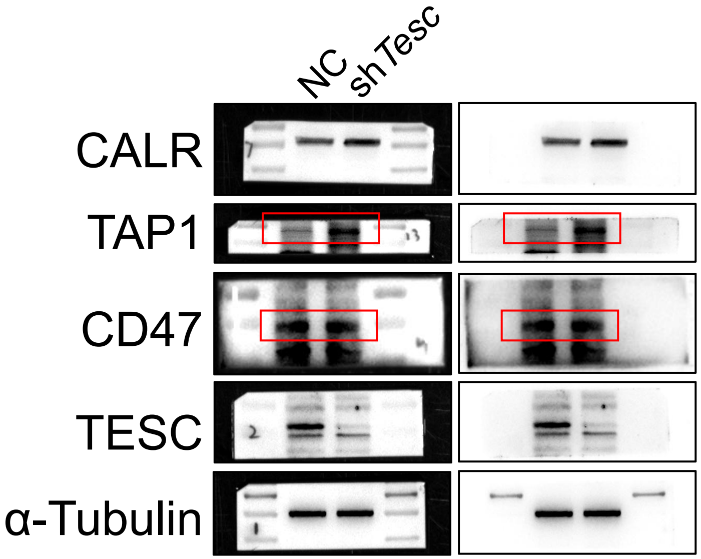

Full unedited blot for Figure S4I

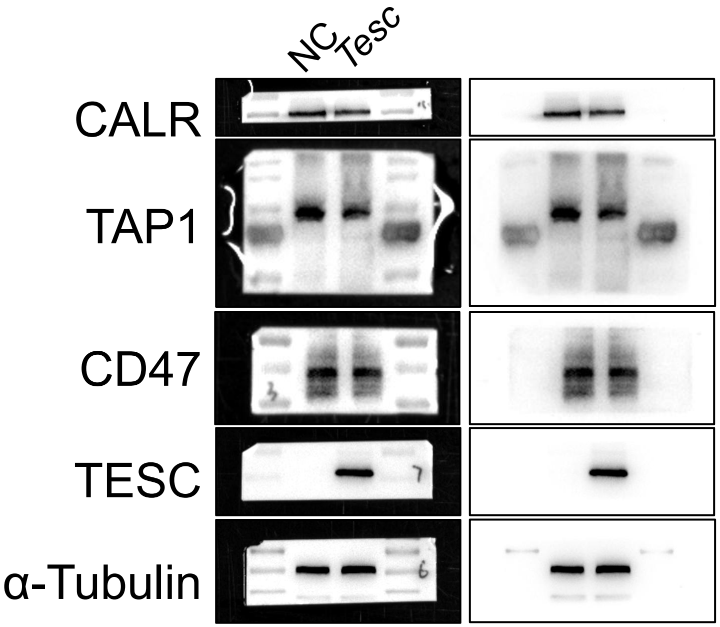

Full unedited blot for Figure S4J

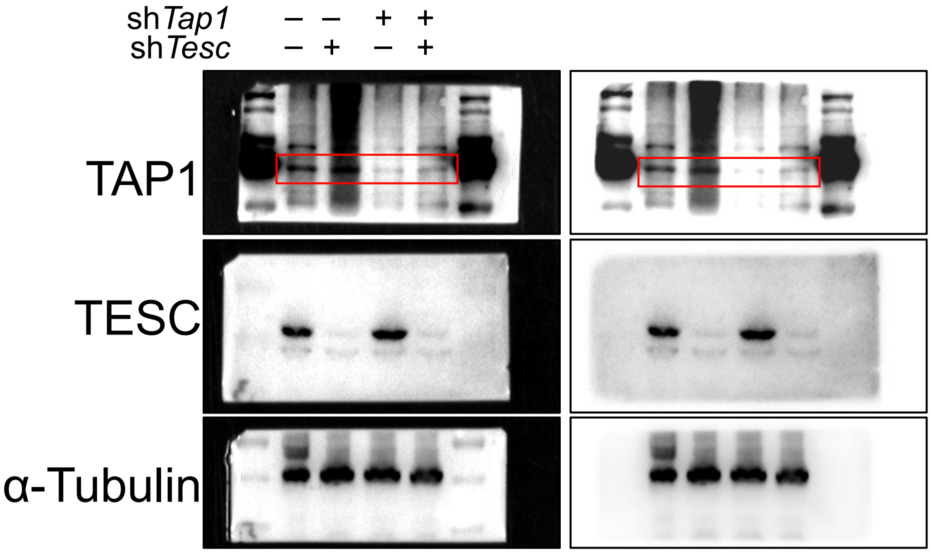

Full unedited blot for Figure S4O

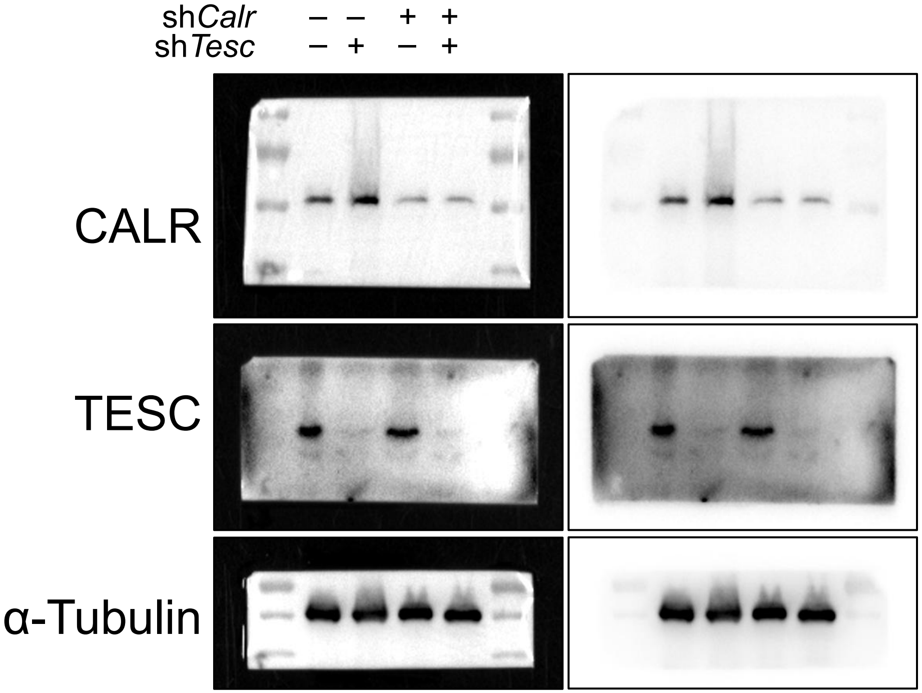

Full unedited blot for Figure S4Q

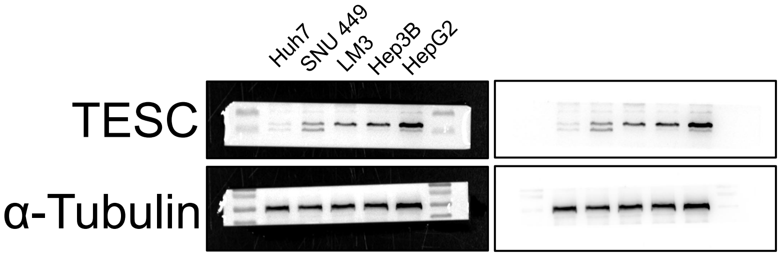

Full unedited blot for Figure S4R

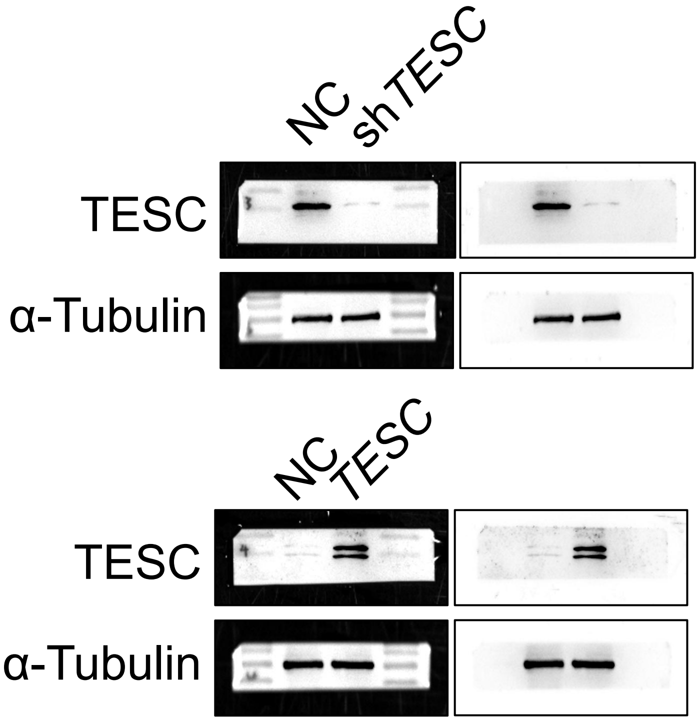

Full unedited blot for Figure S5C

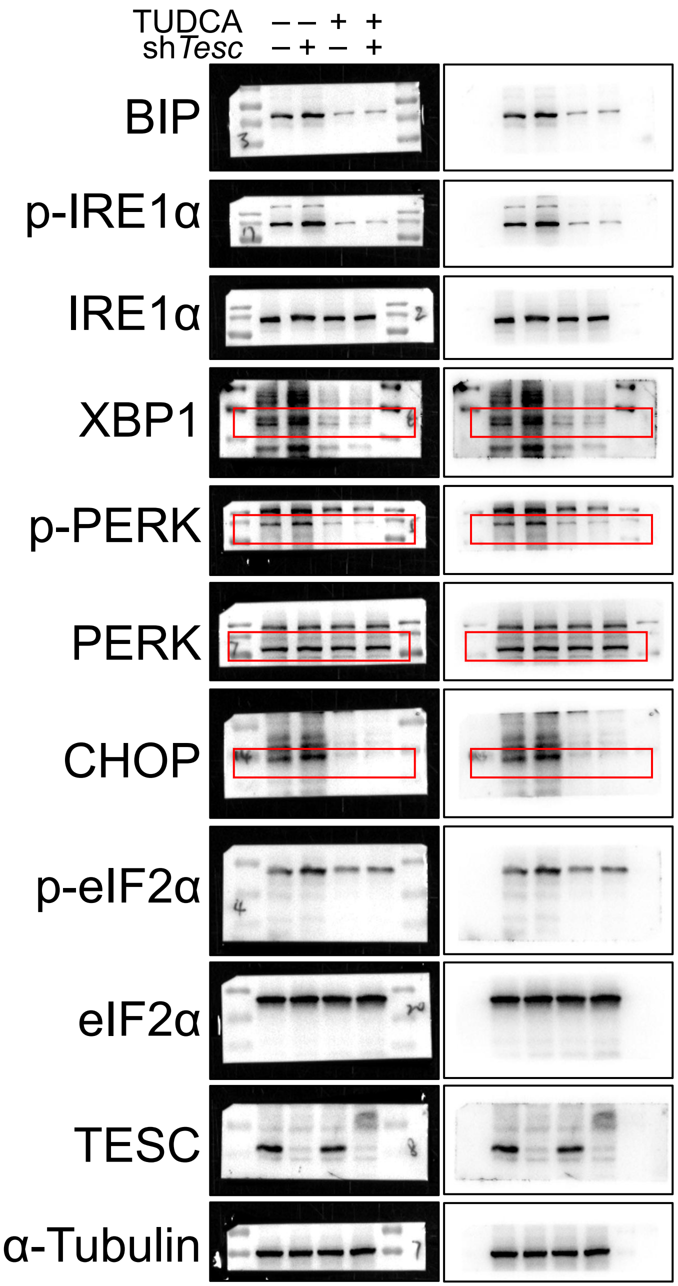

Full unedited blot for Figure S6B

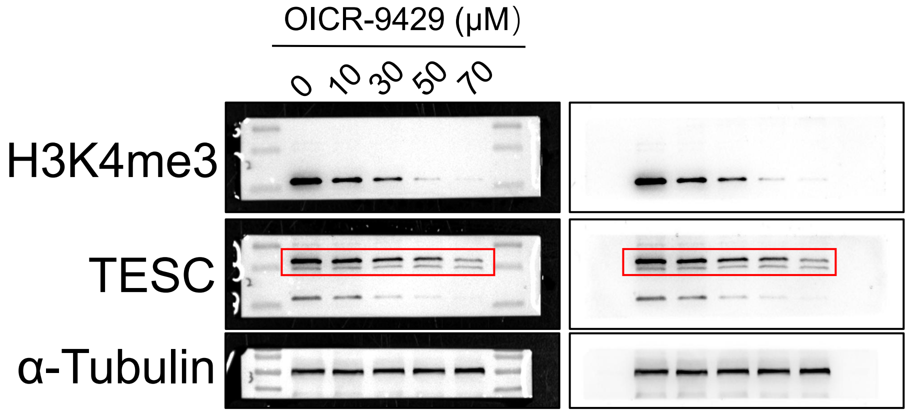

Full unedited blot for Figure S6C

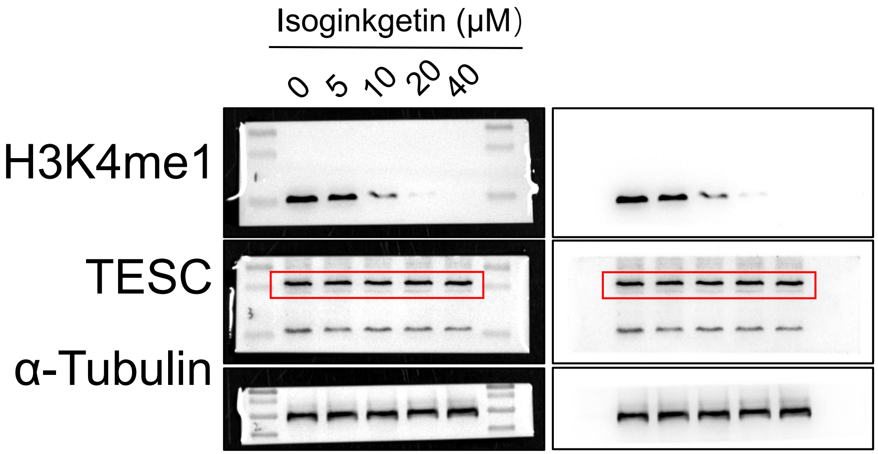

Full unedited blot for Figure S6D

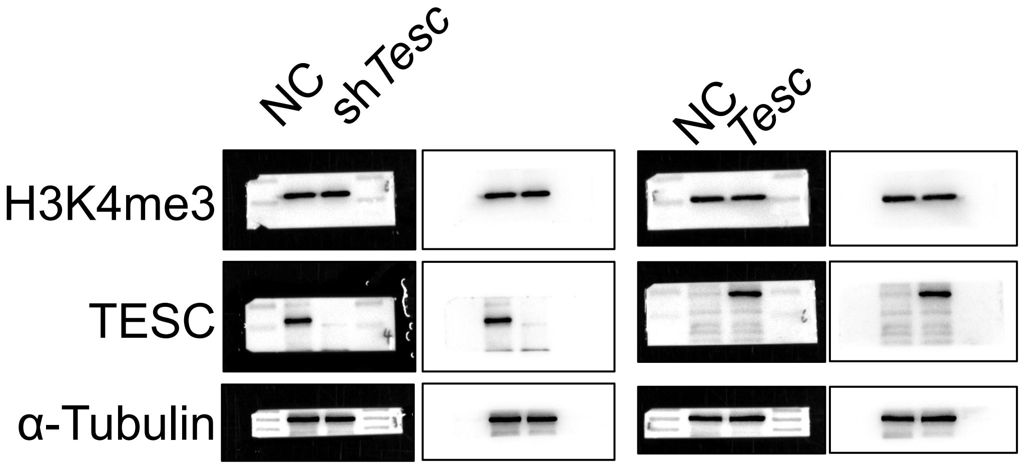

Full unedited blot for Figure S6Q

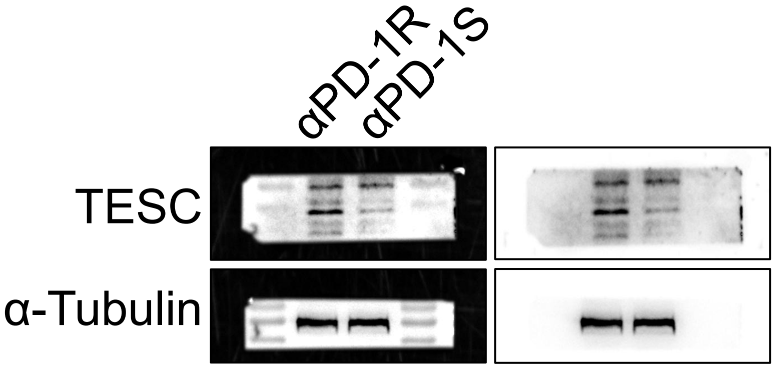

Supplement: Unedited blot and gel images [file jci-136-200415-s134.pdf]
